# Dietary transition to an Indigenous Greenlandic diet induces instant shifts in gut microbiota composition – a pilot intervention study

**DOI:** 10.3389/frmbi.2026.1832705

**Published:** 2026-05-21

**Authors:** Mads B. W. Bjørnsen, Katie M. Bourke, Catherine Stanton, R. Paul Ross, Anders J. Hansen, Aviaja L. Hauptmann

**Affiliations:** 1 SILA Department, Institute of Health and Nature, Ilisimatusarfik – University of Greenland, Nuuk, Greenland; 2 Section for Geogenetics, Globe Institute, University of Copenhagen, Copenhagen, Denmark; 3 Paul Ross Lab, APC Microbiome Ireland, College of Medicine & Health, University College Cork, Cork, Ireland; 4 Teagasc Food Research Institute, Moorepark, Fermoy, Co. Cork, Ireland; 5 Center for Evolutionary Hologenomics, Globe Institute, University of Copenhagen, Copenhagen, Denmark

**Keywords:** Arctic Indigenous foods, dietary intervention, gut microbial diversity, gut microbiota, longitudinal study, non-Western diet, unprocessed food

## Abstract

**Introduction:**

Non-Western diets are increasingly studied for their relationship to gut microbiota composition and diversity, although most research in this area has focused on plant-based, fiber-rich diets. Here, we present a single-participant longitudinal study investigating gut microbiota dynamics during a transition from a Western diet to a 12-week Indigenous Arctic animal-based diet composed of minimally processed raw, dried, and fermented animal-source foods. During one month of this period, the participant consumed dried whole fish (*ammassak*), including intestinal contents, representing a form of gastrophagy, a practice common to the Arctic diet, that may increase exposure to food-associated microbes.

**Methods:**

Fecal samples (n = 29) were collected before, during, and after the Arctic diet phase. 16S rRNA gene sequencing of the V3-V4 region was used to profile bacterial communities. Diversity metrics, Firmicutes/Bacteroidota (F/B) ratios, and taxonomic composition analyses were performed to assess compositional shifts across diet phases.

**Results:**

Alpha diversity remained relatively steady throughout the study, with a tendency toward higher values during the Arctic diet. The F/B ratio increased from 1.31 to 2.12 during the Arctic diet phase and remained elevated (2.38) after returning to a Western diet. Beta diversity analysis revealed significant restructuring of the gut microbiota at the onset of the Arctic diet, followed by partial reversibility upon returning to a Western diet. Fiber-associated taxa like *Prevotella* 9 disappeared, and *Bifidobacterium* declined, while protein- and fat-associated taxa, including *Bacteroides*, *Lachnoclostridium*, and *Alistipes*, increased. Several genera appeared during the Arctic diet phase that were absent during the preceding Western diet phase, consistent with altered microbial exposure. Among those, *Photobacterium* was also detected in the ammassak, suggesting potential microbial exposure during the gastrophagy period.

**Discussion:**

These results provide preliminary evidence that the gut microbiota can shift substantially during an Indigenous Arctic dietary transition. Because the Arctic diet also substantially overlapped with sustained high physical activity, the observed changes should be interpreted in the context of a combined dietary and lifestyle transition. These findings highlight the need for a better understanding of underrepresented dietary patterns, such as those of Arctic Indigenous communities, and their relationship with the gut microbiota.

## 1 Introduction

The human gut microbiota is a complex and dynamic ecosystem that contributes to multiple aspects of host physiology, including immune regulation, nutrient metabolism, and gut-brain axis signaling (Gilbert et al., 2018). Among the many environmental factors that shape the gut microbiome, diet is considered one of the most influential (Turnbaugh et al., 2009). Both short- and long-term changes in dietary intake have been shown to modulate gut microbial composition and diversity, often leading to measurable changes within days (Wu et al., 2011; David et al., 2014; Leeming et al., 2019).

In recent decades, populations in industrialized Western societies have experienced a marked decline in gut microbial diversity (Sonnenburg and Sonnenburg, 2019), a trend increasingly linked to rising rates of chronic diseases, such as inflammatory diseases and metabolic disorders (Leeming et al., 2019; Sonnenburg and Sonnenburg, 2019). This loss of microbial diversity has been linked to dietary changes, characterized by low fiber intake and high consumption of ultra-processed foods. And also, the widespread use of antibiotics and diminished exposure to environmental microbes due to high sanitation standards, and limited food microbial complexity (Wastyk et al., 2021).

While most human microbiome research has focused on Western dietary patterns (Heiman and Greenway, 2016) and disproportionately reflects industrialized populations (Sonnenburg and Sonnenburg, 2019), growing attention is being devoted to non-Western diets, many of which are associated with distinct patterns of microbial exposure and gut microbial composition (De Filippo et al., 2010; Martínez et al., 2015; Smits et al., 2017). Diets of particular interest include fresh, whole foods with minimal processing, particularly when incorporated into foodways that expose individuals to a broad range of environmental microbes (Sonnenburg and Sonnenburg, 2019). Many of these diets are rich in plant-based fibers and have been consistently associated with elevated gut microbial richness and a predominance of fiber-degrading taxa, such as *Prevotella* and *Ruminococcus* (De Filippo et al., 2010; Schnorr et al., 2014; Martínez et al., 2015). Recent work has shown that short-term consumption of exclusively wild foods can induce large-scale alterations in the human gut microbiota, with some changes remaining partially persistent after the intervention ends (Rampelli et al., 2025).

Far less is known about non-Western animal-based diets, which remain underrepresented in microbiome studies. While dietary fibers are important, recent work highlights that proteins and fats also shape the gut microbiota, and their effects are highly context-dependent (Leeming et al., 2021). Arctic Indigenous communities provide a distinct example of such animal-based dietary cultures, relying primarily on meat, fat, and organs, with minimal consumption of plants and dairy (Kuhnlein and Receveur, 2007). In addition to their nutritional composition, these diets may also differ from Western industrialized diets in the types of microbes associated with minimally processed foods. Traditionally prepared Inuit foods in Greenland have been shown to harbor more diverse microbial composition than their industrial counterparts (Hauptmann et al., 2020). While fermented plants and dairy are well-known sources of dietary microbial exposure, Indigenous Arctic food systems may also involve less-studied microbial exposures through the consumption of raw animal foods, gastrointestinal contents, and fermented animal-based foods (Nansen, 1891; Eidlitz, 1969).

One distinctive feature of some Indigenous Arctic food practices is gastrophagy, that is, the consumption of the intestines and their contents (Nansen, 1891; Eidlitz, 1969). From a microbiological perspective, this practice may represent a route of exposure to microbes associated with the animal gut and the food matrix, but this possibility remains largely unexplored, as the main focus has been on taste, culture, and nutrition (Buck and Stringer, 2014; Buck et al., 2016). A relevant example of gastrophagy is the consumption of whole, dried capelin (*Mallotus villosus*) ammassak in Greenlandic, a small fish that plays a central role in Arctic Indigenous food culture (Nansen, 1891; Eidlitz, 1969; Berthelsen, 1935). It is one of the most common fish species identified through DNA investigations of Thule Inuit kitchen middens (Seersholm et al., 2022). Ammassak are caught during predictable spawning migrations and traditionally dried on rocks or bushes for winter storage. The dried fish are traditionally consumed whole, including their intestines and their contents. Recent work integrating microbiological analysis and Indigenous knowledge has demonstrated that dried ammassak harbor diverse microbial communities (Hauptmann et al., 2020), making ammassak a relevant food item for exploring food-associated microbial exposure. This practice of consuming the entire animal, including its gastrointestinal contents, may increase exposure to food-associated microbes derived from both the fish itself and its gastrointestinal contents (Hauptmann et al., 2020).

Existing data on the Inuit gut microbiome are extremely limited, and only two studies have examined how Inuit gut microbial communities differ from Westernized microbiomes in relation to diet (Dubois et al., 2017; Girard et al., 2017). However, neither study examined the dynamics of the gut microbiota during strict adherence to an Indigenous Arctic diet. Rather, “traditional” dietary patterns were evaluated in a mixed dietary setting, where Indigenous foods were consumed alongside Western foods and varied among individuals and communities. Participants were classified as following a traditional diet based on frequent intake of raw or minimally processed game, particularly marine mammals, even though they also consumed imported, packaged, or otherwise Westernized foods. As a result, these studies could not isolate gut microbiota patterns associated with a strictly Indigenous Arctic animal-based diet or capture within-subject changes during the transition from a Western diet. To our knowledge, no previous longitudinal study has followed gut microbiota dynamics during such a dietary transition.

In this study, we investigate the impact of a dietary shift from a Western to a non-Western diet, specifically adopting an Arctic diet rich in animal-based and minimally processed foods, on the human gut microbiota. The dietary shift lasted 12 weeks, exceeding the duration of many typical short-term nutritional interventions, which generally ranges from days to a few weeks, and was sufficient to observe substantial and potentially stable changes in the microbial community (David et al., 2014; Leeming et al., 2019). Using a single-participant longitudinal intervention design, we tracked gut microbial composition before, during, and after the dietary shift. This included a one-month ammassak intervention phase during the Arctic diet, allowing us to assess the temporal dynamics and short-term persistence of microbial change and to explore potential overlap between ammassak-associated and gut-associated taxa. We hypothesized that the transition from a Western diet to an Indigenous Arctic animal-based diet would be associated with restructuring of the gut microbiota, and that some of these changes would persist after return to the Western diet. We further hypothesized that consumption of whole ammassak, including intestinal contents, as a form of gastrophagy, would coincide with the appearance or increased relative abundance of gut taxa that overlapped with those detected in the ammassak intestines, consistent with potential food-associated microbial exposure. More broadly, because the intervention included minimally processed raw, dried, and fermented animal-source foods, we expected the Arctic diet to be associated with compositional changes consistent with distinct food-associated microbial exposure, including the appearance or increased abundance of taxa not detected during the initial Western diet baseline period. Because the Arctic diet also overlapped with sustained physical activity during a coastal qajaq expedition, the observed changes should not be interpreted as effects of diet in isolation, but in the context of these concurrent exposures.

By focusing on a dietary tradition rarely included in microbiome research, this study contributes to a more specific understanding of gut microbiota dynamics in an underrepresented Arctic dietary context.

## 2 Materials and methods

### 2.1 Ethical statement

All procedures involving human samples were approved by Den Videnskabsetiske Udvalg for Grønland (The Scientific Ethics Committee for Greenland), case no. 2023-23683. The participant provided written informed consent permitting the use of data for research purposes; confidentiality safeguards were applied throughout data handling and reporting.

### 2.2 Sampling

#### 2.2.1 Fecal sampling

**Figure 1:**
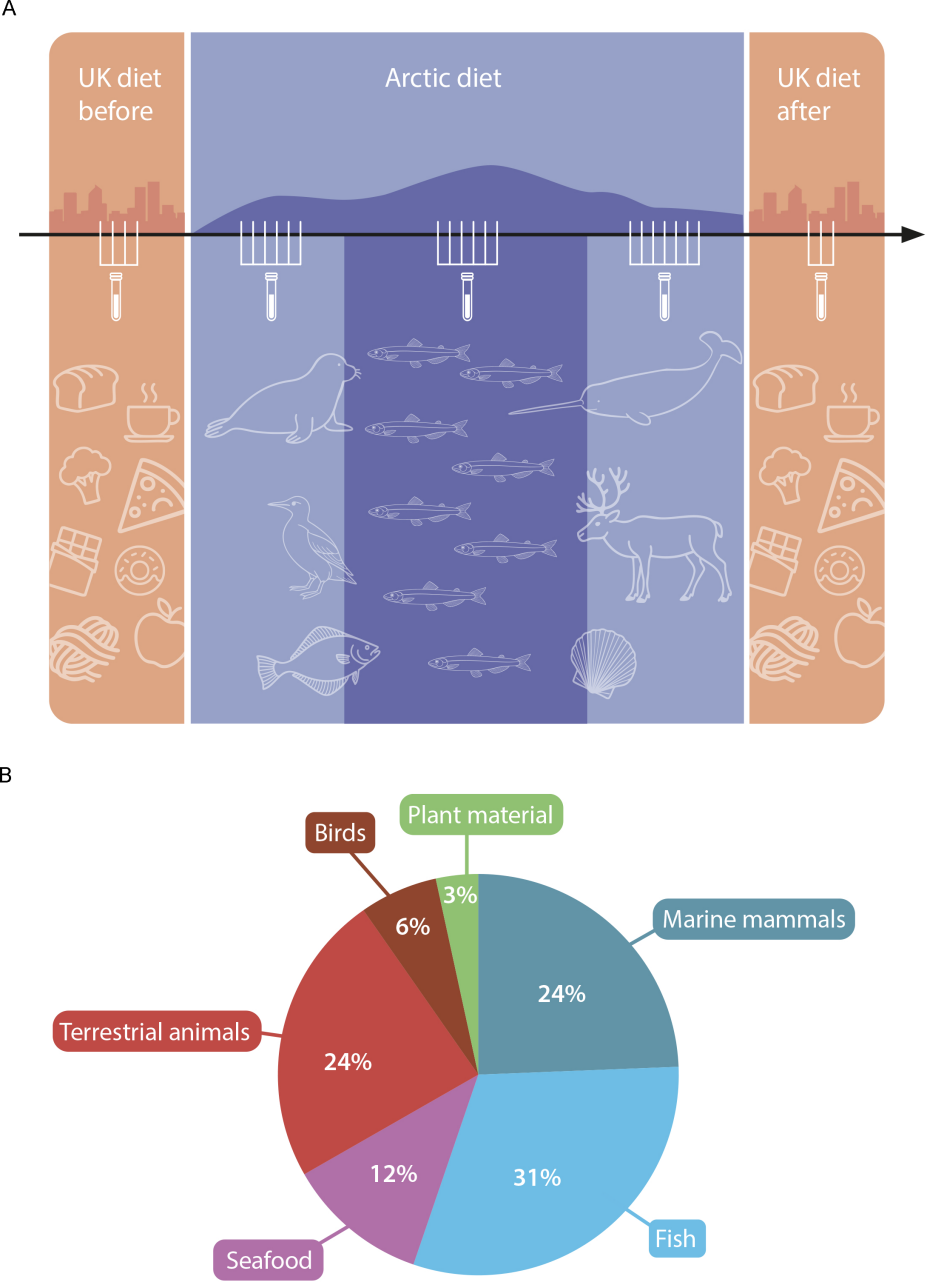
Study design for a longitudinal single-participant dietary intervention and composition of foods consumed during the Arctic diet. **(A)** Schematic overview of the study comprising three main dietary phases: the UK diet before, the Arctic diet, and the UK diet after. The Arctic diet period overlapped substantially with the qajaq expedition and sustained high physical activity. This period was further divided into three subphases: pre-ammassak, ammassak-intervention, and post-ammassak. During the Arctic diet phase, the participant consumed locally sourced Arctic foods, including marine mammals, terrestrial mammals, fish, seafood, birds, and small amounts of plant material. Fecal samples were collected repeatedly across all phases (sampling icons) to track changes in gut microbial composition. The figure includes only samples retained after bioinformatic quality control, comprising 4 samples in the UK diet before, 19 samples across the Arctic subphases (6 pre-ammassak, 6 ammassak-intervention, and 7 post-ammassak), and 3 samples in the UK diet after. The UK diet phases reflect a Western dietary pattern characterized predominantly by processed foods, whereas the Arctic diet phase reflects a primarily animal-based dietary pattern on locally sourced Arctic foods. **(B)** Proportional breakdown of all foods consumed during the Arctic diet phase, based on detailed dietary records. The diet was dominated by fish (light blue, 31%), marine mammals (dark blue, 24%), and terrestrial mammals (red, 24%), with smaller contributions from seafood (purple, 12%), birds (brown, 6%), and plant material (green, 3%). These proportions illustrate the primarily animal-based composition of the Indigenous Arctic diet consumed during the intervention.

The participant was a 53-year-old adult male. Before the expedition, the participant reported consuming his self-reported typical UK-based Western diet, which he described as similar before and after the qajaq expedition. Example diet records from several days indicated a mixed omnivorous pattern including bread, rice, dairy products, eggs, meat, offal, fish, chocolate, coffee, and some plant foods (**Supplementary Table 1**). He also reported minimal alcohol consumption outside the study period, but no alcohol, smoking, or antibiotic use during the study period. Baseline measurements showed a body mass index (BMI) of 26.4 kg/m^2^, a body fat mass of 18.3 kg, a body fat percentage of 20.3%, and an estimated basal metabolic rate of 2083 kcal/day. Fecal samples were collected twice weekly in a longitudinal dietary intervention study design conducted in 2023. The participant transitioned from a Western diet in the United Kingdom to a strictly Indigenous Arctic animal-based diet in Kalaallit Nunaat (Greenland) for approximately 12 weeks (April 15th to July 7th). The Arctic diet consisted exclusively of locally sourced foods and was composed primarily of meat and fat, including seal meat and fat, ammassak, halibut, cod, bird eggs, caribou, and muskox (**Figure 1**). During this period, no Western food was consumed. From April 26th to July 7th, the participant undertook a coastal qajaq expedition along Greenland’s west coast from south to north, resulting in sustained, high physical activity throughout this period. In this study, the term Arctic diet refers to the dietary intervention period during which the participant consumed a strictly Indigenous Arctic animal-based diet. Because this interval overlapped substantially with the qajaq expedition and sustained high physical activity, microbiota changes observed during the Arctic diet period cannot be attributed to diet alone and are interpreted in the context of these concurrent exposures. Western foods were reintroduced after July 7th.

The Arctic diet period was divided into three subphases: an initial phase without ammassak (pre-ammassak; April 15th to May 14th), a phase during which ammassak was included in the diet (ammassak-intervention; May 15th to June 15th), and a final phase where ammassak was again excluded (post-ammassak; June 16th to July 7th). This subdivision was used to distinguish samples collected before, during, and after regular ammassak consumption while the participant otherwise remained on the Arctic diet. A full breakdown of daily dietary intake is provided in **Supplementary Table 2**.

A total of 29 fecal samples were collected over the three study phases: four during the UK diet before the dietary shift (UK diet before), 21 during the Arctic diet phase (seven pre-ammassak consumption, six during ammassak-intervention, and eight post-ammassak consumption), and four after returning to the UK diet where he gradually introduced processed foods to his diet again (UK diet after). Prior to the expedition launch, sterile 5-mL Eppendorf tubes pre-filled with 3 mL of RNAlater^®^ (Thermo Fisher Scientific, Massachusetts, United States) were prepared for fecal sample collection. Upon sampling, fecal material was placed directly into the tubes. Tubes containing RNAlater^®^ only were carried alongside the samples throughout the expedition and used as negative controls. As the participant reached settlements along the expedition route, samples were shipped to Ilisimatusarfik – The University of Greenland in Nuuk, where they were stored at -20 °C until further processing.

#### 2.2.2 Ammassak sampling

A total of 18 swab samples of gut contents from dried ammassak were obtained from a local producer in the Disko Bay area of West Greenland and collected under field conditions. Sampling was performed using sterile gloves, and each swab was placed in a sterile 5-mL Eppendorf tube containing 3 mL of RNAlater^®^ (Thermo Fisher Scientific, Massachusetts, United States). Tubes containing RNAlater^®^ only were included as negative controls during sampling. Samples were shipped to Ilisimatusarfik and stored at -20 °C until further processing.

### 2.3 Sample processing

#### 2.3.1 DNA extraction and 16S rRNA gene sequencing

All samples were processed at the APC Microbiome Ireland laboratory (Cork, Ireland). DNA was extracted from 0.2g of stool sample using the repeated bead-beating plus column method as described by Yu and Morrison (2004) and Ghosh et al. (2020), with minor modifications, including an extraction blank to monitor potential contamination. Nucleic acids were purified using the QIAmp Fast DNA Stool Mini Kit (Qiagen, UK), and DNA was stored at -20 °C. DNA concentration was quantified using a Qubit fluorometer (Life Technologies Corp., Carlsbad, CA).

Amplicon libraries were prepared according to the Illumina 16S Metagenomic Sequencing Library Preparation protocol, targeting the V3-V4 region of the 16S rRNA gene. PCR amplification was performed using Illumina adapter-tailed primers: Forward: 5’-TCGTCGGCAGCGTCAG

ATGTGTATAAGAGACAGCCTACGGGNGGCWGCAG and Reverse: 5’-GTCTCGTGG

GCTCGGAGATGTGTATAAGAGACAGGACTACHVGGGTATCTAATCC. The PCR amplification was carried out with KAP HiFi HotStart ReadyMix (Roche, Switzerland) using the following conditions: initial denaturation at 95 °C for 3 min; 25 cycles of 95 °C for 30 sec, 55 °C for 30 sec, and 72 °C for 30 sec; final extension at 72 °C for 5 min.

A subsequent eight-cycle Index PCR was performed to attach dual indices using the Nextera XT Index kit v2 Set C (Illumina, San Diego, CA, USA). PCR products were visualized on a 1.5% agarose gel stained with SYBR Safe (Thermo Fisher, MA, USA), purified with AMPure XP beads (Beckman Coulter, CA, USA), and quantified using the Qubit 1X dsDNA HS Assay Kit (Thermo Fisher). Fragment size distributions were confirmed on an Agilent 4200 TapeStation (Agilent Technologies, Santa Clara, CA, USA). Libraries were normalized to 20 nM, pooled in equal volumes (20 µL per sample), and sequenced at Genewiz (Leipzig, Germany) on an Illumina MiSeq platform (2 x 300 bp paired-end reads).

Of the 18 ammassak samples collected, 7 did not yield sufficient DNA for downstream sequencing and were therefore excluded from sequencing.

#### 2.3.2 Bioinformatic preprocessing and data filtering

Raw sequences were processed in RStudio v.4.3.1 using the DADA2 pipeline v.1.28.0 (Callahan et al., 2016), which performs quality filtering, dereplication, denoising, merging of paired-end reads, and chimera removal. For the DADA2 pipeline, the standard settings were used, except for the filterAndTrim function, where trimLeft = c(18, 21) was applied to remove the V3-V4 primer sequences from the forward and reverse reads. Taxonomic amplicon sequence variant (ASV) classification was performed against the SILVA reference database v.138.1 (Quast et al., 2013). The resulting ASV tables, taxonomic classification, and sample metadata were imported into phyloseq v.1.52.0 (McMurdie and Holmes, 2013) for downstream analysis. Negative controls were processed alongside biological samples to monitor potential contamination during downstream laboratory procedures. During DADA2 preprocessing, negative controls were filtered out because they had too few reads. Taxa lacking Kingdom-level classifications were removed (29 ASVs excluded). To ensure data quality and comparability, samples with fewer than 2,500 reads were excluded from further analysis, resulting in the removal of 3 human samples (2 samples from the Arctic diet and 1 sample from the UK after) (Callahan et al., 2016; Weiss et al., 2017; Prodan et al., 2020; Nearing et al., 2022). Low-abundance ASVs (<5 reads total) were removed to reduce spurious diversity (McMurdie and Holmes, 2014; Callahan et al., 2017; Knight et al., 2018; Willis, 2019). This step removed 306 ASVs from the human dataset and 128 ASVs from the ammassak dataset. Filtering was performed independently on the human and ammassak datasets prior to downstream analyses of diversity and composition. After filtering, the human dataset comprised 26 fecal samples, and the ammassak dataset comprised 11 samples.

#### 2.3.3 Analyzing and visualization

Before composition-based analyses, data were transformed to relative abundances using total sum scaling. Given the single-participant longitudinal design, statistical analyses were used to assess within-subject temporal differences across phases and were interpreted as exploratory. Because the qajaq journey and associated high physical activity overlapped substantially with the Arctic diet phase, physical activity and expedition-related environmental change were treated as concurrent exposures and were not analyzed as independent variables. Given the temporal overlap between these factors and the Arctic diet phase, their effects could not be statistically separated from those of diet. The Arctic diet was therefore treated analytically as a combined exposure period rather than a diet-only intervention. Analyses were performed at two levels: first, across the three main study phases (UK diet before, Arctic diet, and UK diet after), and second, within the Arctic diet across the three subphases (pre-ammassak, ammassak-intervention, and post-ammassak), to assess whether microbiota patterns differed across the Arctic subphases, including the ammassak-intervention period. Alpha diversity was assessed using four metrics: Observed richness, Faith’s Phylogenetic Diversity (PD), Shannon Index, and Simpson Index. The Observed, Shannon, and Simpson indices were calculated using the R packages *phyloseq* v.1.52.0 (McMurdie and Holmes, 2013) and *microbiome* v.1.30.0 (Lahti and Shetty, 2017), while Faith’s PD was computed using picante v.1.8.2 (Kembel et al., 2010).

For the human dataset, the Shapiro-Wilk test was applied to assess normality. ANOVA was used where assumptions were met. Otherwise, Kruskal-Wallis tests were applied. Where pairwise comparisons were required, Wilcoxon rank-sum tests were used, with Benjamini–Hochberg correction applied. These tests were applied both to comparisons across the main diet phases and, where relevant, to comparisons across the three Arctic subphases. Beta diversity was quantified using Bray-Curtis dissimilarities and visualized using Non-metric Multidimensional Scaling (NMDS). Group differences were assessed using PERMANOVA (999 permutations) using the R package vegan v.2.7.3 (Oksanen et al., 2019), and pairwise comparisons were performed using the R package pairwiseAdonis v.0.4.1 (Martinez Arbizu, 2020). Differentially abundance analysis was used to identify bacterial genera that changed significantly between diet phases, using the R package ANCOMBC v2.10.1 (Lin and Peddada, 2020, 2024). In parallel, non-parametric Wilcoxon rank-sum tests were conducted for pairwise comparisons with the Benjamini-Hochberg correction and were considered significant at an adjusted *p < 0.05* in both analyses. Microbial patterns were visualized using the R packages ggplot2 v.4.0.3 (Wickham, 2008) and ANCOMBC v.2.10.1 (Lin and Peddada, 2020).

##### 2.3.3.1 Targeted analysis of *Prevotella* clades

To explore the ecological behaviors of different *Prevotella* lineages, we examined clades defined in the SILVA v. 138.1 taxonomy, which separates *Prevotella* into numbered clades (e.g., *Prevotella* 7 and *Prevotella* 9) (Wallen et al., 2020; Larzul et al., 2024), and non-numbered *Prevotella* clades based on phylogenetic divergence. Fourteen ASVs were extracted from the data belonging to the clades *Prevotella* (n = 4)*, Prevotella 7* (n = 5), and *Prevotella 9* (n = 5), which were then exported and queried against the NCBI 16S rRNA database using BLASTn to identify their closest species-level matches. High-confidence hits were defined as those with ≥98% sequence identity corresponding to the V3-V4 region of the 16S rRNA gene.

Of the 14 ASV sequences that were sent to BLAST, eight sequences passed the threshold. For the ASVs classified under *Prevotella* (not assigned to a numbered clade), four passed the criteria and matched cultured species: *Prevotella bivia* (NR_113096.1; 99-100% identity), *Prevotella disiens* (NR_113103.1; 100% identity), and *Prevotella buccalis* (NR_113098.1; 99% identity), which has recently been reassigned to the species *Hoylesella buccalis* (Hitch et al., 2022). For the ASVs assigned to numbered clade *Prevotella* 7, none passed the criteria. For the numbered clade *Prevotella* 9, four ASV sequences passed the criteria and matched to *Prevotella copri* (NR_040877.1; 98-99% identity), which has recently been reassigned to the species *Segatella copri* (Blanco-Míguez et al., 2023; Peng and Tun, 2023). Previous studies have shown that *Prevotella* 9 is often associated with *Prevotella copri* (Tett et al., 2019; Yeoh et al., 2022).

Relative abundance of each clade was quantified across the three main diet phases, and statistical differences between phases were tested using Wilcoxon rank-sum tests with Benjamini-Hochberg correction. Results were visualized using box plots to compare abundance trajectories across the three diet phases.

## 3 Results

We characterized the gut microbiota of a participant transitioning from a Western diet to an Indigenous Arctic animal-based diet during a 12-week coastal qajaq expedition along Greenland’s west coast, from south to north. The microbiota of ammassak consumed as part of the Arctic diet were also characterized.

After preprocessing, the human dataset was comprised of 26 fecal samples and 1,161 ASVs, derived from 3,829,219 paired-end sequencing reads with a mean of 147,278 reads per sample. Taxonomic assignment identified 12 phyla and 175 genera. The 26 samples associated with this dataset were from three dietary phases: the UK diet before (n = 4), the Arctic diet (n = 19), and the UK diet after (n = 3). The ammassak dataset comprised 11 samples and 682 ASVs, derived from 448,555 paired-end sequencing reads, with a mean of 40,777 reads per sample. Taxonomic assignment identified 10 phyla and 122 genera. 

Rarefaction curves indicated that all samples reached an asymptote, confirming sufficient sequencing depth for downstream analysis (data not shown). These curated datasets were used for subsequent analyses of alpha diversity, beta diversity, and to profile taxonomic composition to characterize the gut microbiota patterns across the study phases.

### 3.1 Alpha diversity of the human gut microbiota

**Figure 2:**
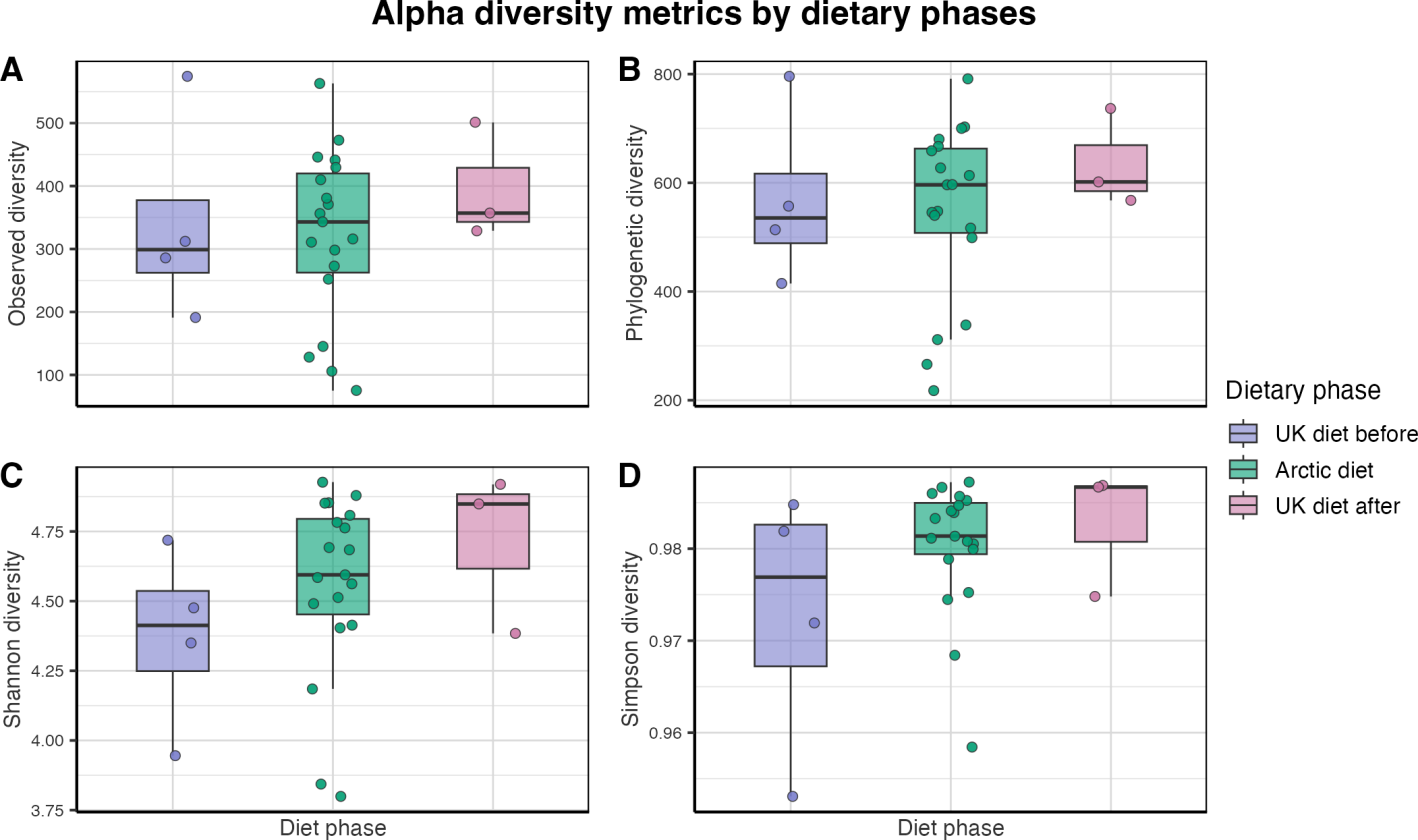
Alpha diversity of the human gut microbiota across dietary phases. Boxplot showing within-sample microbial diversity across 26 fecal samples from three dietary phases: UK diet before (purple, n = 4), Arctic diet (green, n = 19), and UK diet after (pink, n = 3). Points represent individual fecal samples. Four alpha diversity metrics were assessed: **(A)** Observed richness, **(B)** phylogenetic diversity (Faith’s PD), **(C)** Shannon index, and **(D)** Simpson index. Samples from the UK diet phase are shown for descriptive comparison only because of the limited sample size. Statistical comparisons were therefore restricted to the UK diet before and the Arctic diet phases. No significant differences were detected (p > 0.05).

Four alpha diversity metrics, Observed richness, Faith’s Phylogenetic Diversity (Faith’s PD), Shannon diversity index, and Simpson diversity index, were calculated for all 26 human fecal samples spanning the three dietary phases. However, statistical comparisons were restricted to the UK diet before (*n* = 4) and Arctic diet (*n* = 19), as the UK diet after (*n* = 3) contained too few samples for reliable testing; however, they are included in descriptive summaries and visualization to illustrate overall trends across all dietary phases (**Figures 2A–D**). The normality of each metric within dietary groups was assessed using the Shapiro-Wilk test (data not shown). Parametric t-tests were applied for Observed richness and Faith’s PD, which met normality assumptions, while non-parametric Wilcoxon rank-sum tests were used for Shannon and Simpson indices, where normality assumptions were not met in at least one group.

No statistically significant differences were observed between the UK diet before and the Arctic diet for any of the alpha diversity metrics: Observed richness (*p = 0.841*), Faith’s PD (*p = 0.797*), Shannon (*p = 0.209*), and Simpson (*p = 0.351*). To further explore whether microbial diversity varied within the Arctic diet, alpha diversity was compared across the three Arctic subphases: pre-ammassak, ammassak-intervention, and post-ammassak (**Supplementary Figure 1**). No significant differences were observed across the Arctic subphases based on Observed richness (*p = 0.667*), Faith’s PD (*p = 0.540*), Shannon (*p = 0.720*), and Simpson (*p = 0.549*).

### 3.2 Beta diversity of the human gut microbiota

**Figure 3:**
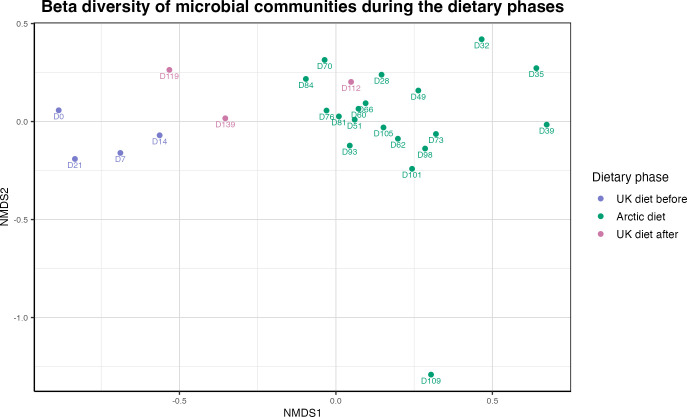
Beta diversity of the human gut microbiota across dietary phases (Bray-Curtis, NMDS). Non-metric multidimensional scaling (NMDS) based on Bray-Curtis dissimilarity illustrating gut microbial community composition across 26 fecal samples from three dietary phases: UK diet before (purple, n = 4), Arctic diet (green, n = 19), UK diet after (pink, n = 3). Samples showed separation by dietary phase, consistent with compositional differences across the dietary phases. PERMANOVA revealed a significant overall difference in microbial composition between phases (R^2^ = 0.289, p = 0.0001), with significant pairwise differences between the UK diet before and Arctic diet phases (R^2^ = 0.261, p = 0.003), and between the Arctic diet and UK diet after phases (R^2^ = 0.105, p = 0.013), and between the two UK diet phases (R^2^ = 0.400, p 0.02). Each point represents the microbial composition of a sample and is labeled with the number of days from the start of the study. Each number represents the day the sample was taken, with D0 (purple) being the first sample day and D139 (pink) being the last sample date.

Beta diversity analyses were performed to assess differences in microbial communities across the three dietary phases: the UK diet before, the Arctic diet, and the UK diet after. Bray-Curtis dissimilarity was used to calculate differences in community composition, and ordination was visualized using Non-Metric Multidimensional Scaling (NMDS) (**Figure 3**). The sample numbers assigned to each sample correspond to the sampling days (D0-D139) and can be cross-referenced with the daily dietary log in **Supplementary Table 2**. The NMDS ordination revealed clustering of microbial communities by dietary phase, with the Arctic diet phase exhibiting distinct separation from the UK diet both before and after the intervention. The stress value for the Bray-Curtis NMDS ordination was 0.113, indicating a good representation of community dissimilarity in two dimensions. PERMANOVA revealed significant differences in microbial community composition between dietary phases, as indicated by Bray-Curtis dissimilarity (*R^²^ = 0.289, p = 0.0001*). Pairwise comparisons based on Bray-Curtis dissimilarity revealed a significant shift in microbial composition between the UK diet before and the Arctic diet (*R^2^ = 0.261, p = 0.003*), as well as between the Arctic diet and the UK diet after (*R^2^ = 0.105, p = 0.013*), and between the UK diet before and the UK diet after (*R^2^ = 0.400, p = 0.02*).

To assess whether community composition also varied within the Arctic diet, beta diversity analysis was performed using Bray-Curtis dissimilarities using only the three Arctic subphases, pre-ammassak, ammassak-intervention, and post-ammassak, and visualized using NMDS (**Supplementary Figure 2**). PERMANOVA showed significant differences in microbial community composition across the three Arctic subphases (*R^²^ = 0.210, p = 0.0002*). Pairwise comparisons showed significant differences between pre-ammassak and ammassak-intervention (*R^²^ = 0.179, p = 0.015*), pre-ammassak and post-ammassak (*R^²^ = 0.164, p = 0.004*), and ammassak-intervention and post-ammassak (*R^²^ = 0.156, p = 0.01*).

### 3.3 Changes in taxonomic composition across dietary phases

**Figure 4:**
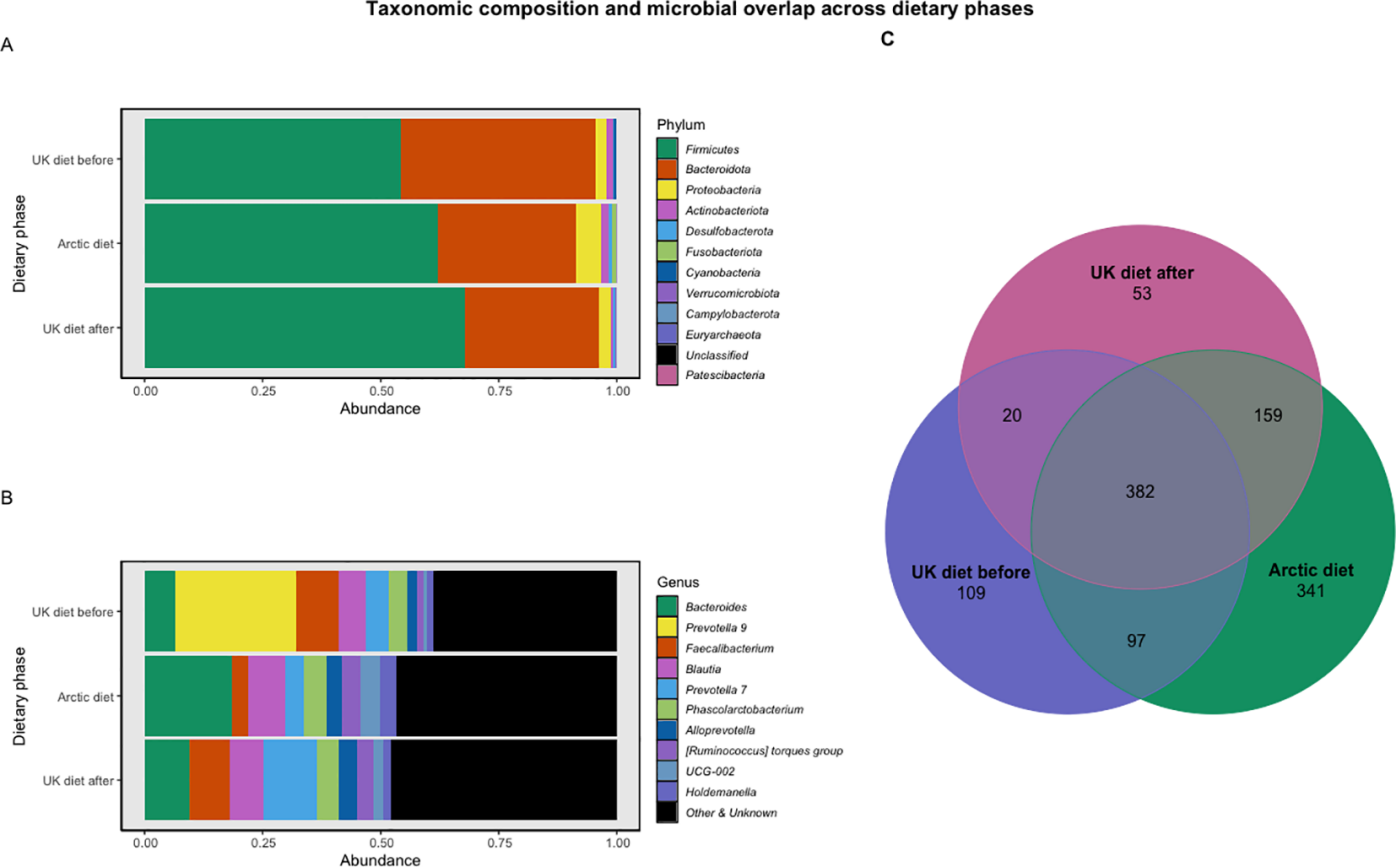
Relative abundance of dominant bacterial taxa and ASV overlap across dietary phases. **(A)** Mean relative abundance of dominant bacterial phyla across the three dietary phases: UK diet before (n = 4), Arctic diet (n = 19), and UK diet after (n = 3). **(B)** Mean relative abundance of dominant bacterial genera across the three dietary phases. At the genus level, taxa not among the top 10 abundant genera are grouped under “Other & Unknown”. **(C)** Venn diagram showing the number of shared and unique ASVs across dietary phases. The Arctic diet exhibited the highest number of unique ASVs (n = 341), followed by the UK diet before (n = 109) and the UK diet after (n = 53), with 382 ASVs shared across all three phases.

The mean relative abundance and standard deviation (SD) of the five most dominant phyla and genera were calculated for each dietary phase to provide an overview of microbial composition (**Figures 4A, B**).

During the UK diet before phase, the most abundant phyla were *Firmicutes* (54.25% ± 15.78%) and *Bacteroidota* (41.29% ± 16.85%), with a *Firmicutes/Bacteroidota* ratio of 1.31. Other abundant phyla included *Proteobacteria* (2.36% ± 0.93%), *Actinobacteriota* (1.26% ± 0.52%), and *Cyanobacteria* (0.72% ± 1.18%). At the genus level, the microbiota was dominated by *Prevotella 9* (25.68% ± 14.71%), *Faecalibacterium* (9.10% ± 3.24%), a group of unclassified genera (6.76% ± 3.51%), *Bacteroides* (6.40% ± 2.59%), and *Blautia* (5.61% ± 3.62%).

During the Arctic diet phase, *Firmicutes* (62.05% ± 11.92%) remained the most abundant phylum, followed by *Bacteroidota* (29.33% ± 10.62%), with an increased *Firmicutes/Bacteroidota* ratio of 2.12. Other dominant phyla included *Proteobacteria* (5.19% ± 3.66%), *Actinobacteriota* (1.75% ± 2.20%), and *Fusobacteriota* (0.93% ± 2.88%). At the genus level, *Bacteroides* (18.39% ± 8.42%) was the most abundant, followed by unclassified genera (8.55% ± 3.1%), *Blautia* (7.88% ± 4.9%), *Phascolarctobacterium* (4.83% ± 2.11%), and *UCG-002* (4.17% ± 2.67%).

For the UK diet after phase, *Firmicutes* (67.75% ± 3.42%) continued to dominate the phylum-level composition, followed by *Bacteroidota* (28.49% ± 4.52%), with a *Firmicutes/Bacteroidota* ratio further increasing to 2.38. Other dominant phyla included *Proteobacteria* (2.54% ± 0.71%), *Actinobacteriota* (0.45% ± 0.09%), and *Desulfobacterota* (0.35% ± 0.31%). At the genus level, the most abundant taxa were *Prevotella 7* (11.35% ± 7.31%), *Bacteroides* (9.56% ± 0.97%), *Faecalibacterium* (8.4% ± 4.6%), *Blautia* (7.23% ± 0.86%), and a group of unclassified genera (6.71% ± 1.77%).

To assess microbial overlap across dietary phases, a Venn diagram was generated based on shared ASVs (**Figure 4C**). The Arctic diet exhibited the highest number of unique ASVs (n = 341), compared to the UK diet before (n = 109) and the UK diet after (n = 53). Among all phases, 382 ASVs were shared across the three dietary groups. Additionally, 97 ASVs were shared exclusively between the UK diet before and the Arctic diet, 159 between the Arctic diet and the UK diet after, and only 20 between the UK diet before and the UK diet after.

### 3.4 Differential abundance of gut microbial taxa across diets

**Figure 5:**
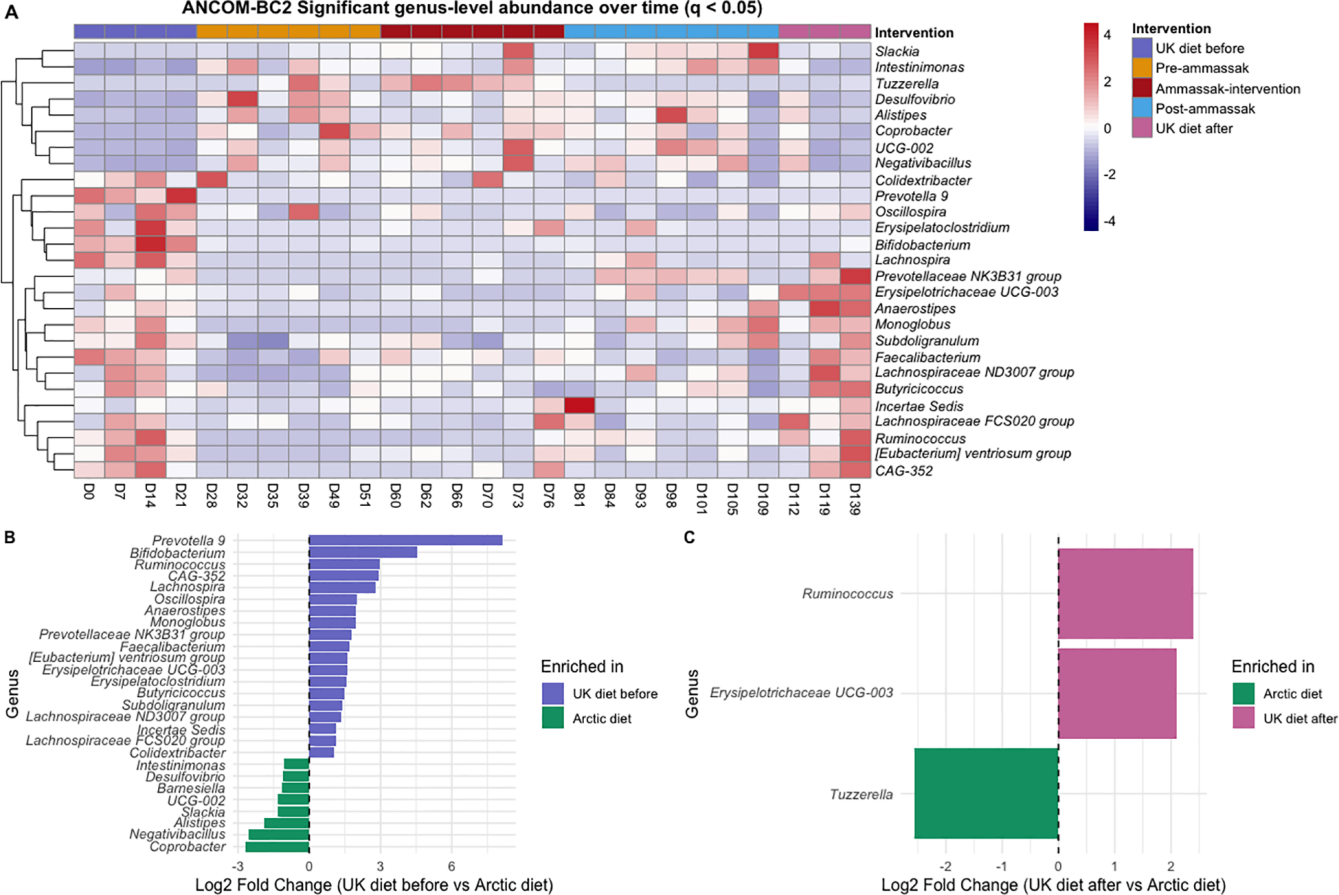
Differentially abundant bacterial genera across the dietary phases. **(A)** Heatmap showing bacterial genera identified as significantly differentially abundant across dietary phases by ANCOM-BC2 (q < 0.05). Columns represent individual fecal samples ordered by study day (n = 26). The colored annotation bar above indicates dietary subphases: purple = UK diet before (n = 4), orange = pre-ammassak (n = 6), red = ammassak-intervention (n = 6), blue = post-ammassak (n = 7), pink = UK diet after (n = 3). The color intensity in the heatmap represents centered and scaled relative abundance (Z-scores), with red indicating higher and blue indicating lower abundance relative to the genus mean. **(B, C)** Bar plots showing ANCOM-BC2-estimated log_2_ fold changes for genera that differed significantly between dietary phases (q < 0.05). In panel **(B)**, positive values indicate enrichment in the UK diet before phase, and negative values indicate enrichment in the Arctic diet. In panel **(C)**, positive values indicate enrichment in the UK diet after phase, and negative values indicate enrichment in the Arctic diet.

To evaluate changes in gut microbial composition across the study phases, we identified bacterial genera that either appeared or disappeared across the dietary phases. Based on ANCOM-BC2 differential-abundance analysis (q < 0.05), a total of 9 genera appeared following the shift from the UK diet to the Arctic diet. In comparison, 19 genera declined or became undetectable during the Arctic diet. No significant difference was observed between the UK diet before and the UK diet after. The heatmap (**Figure 5A**) illustrates the temporal dynamics of these significant genera across all dietary subphases. The bar plots (**Figures 5B, C**) display the log_2_ fold changes between the dietary contrasts.

To complement these findings, a heatmap based on presence/absence analysis (**Supplementary Figure 3**) was used to track genera that appeared or disappeared across the dietary phases. Initially, 47 genera were identified as appearing during the Arctic diet phase. After applying a detection threshold of ≥ 0.1% relative abundance and requiring ≥ 2 samples in this phase, 27 genera were retained as consistently appearing, while seven genera disappeared (**Supplementary Table 3**). Among the taxa that appeared during the Arctic diet, *Slackia*, *Lachnospiraceae UCG-*010, *Family XII UCG-001*, and *the (Eubacterium) nodatum group* first appeared during the Arctic diet, while four genera, *Turicibacter*, *Photobacterium*, *Coprobacillus*, and *Robinsoniella*, first appeared after the ammassak-intervention. The genera that disappeared included *Prevotella 9*, *Agathobacter*, and *Bifidobacterium*.

Pairwise Wilcoxon rank-sum tests revealed several significant differences in the microbial composition at both the phylum and genus levels. At the phylum level, *Cyanobacteria* were significantly less abundant during the Arctic diet compared to the UK diet, both before (*p < 0.001*) and after (*p < 0.001*) the intervention. *Desulfobacterota* was significantly more abundant during the Arctic diet than during the UK diet before (*p = 0.02*).

At the genus level, several taxa were significantly more abundant in the UK diet before. These included *Prevotella* 9 clade (ANCOM-BC *log_2_FC = 8.35*, *q < 0.0001*; Wilcoxon *p = 0.001*), *Bifidobacterium* (ANCOM-BC2 *log_2_FC = 4.64*, *q < 0.0001*; Wilcoxon *p = 0.004*), *Lachnospira* (ANCOM-BC2 *log_2_FC = 2.78*, *q = 0.0015*; Wilcoxon *p = 0.008*), *Ruminococcus* (ANCOM-BC2 *log_2_FC = 2.76*, *q = 0.0012*; Wilcoxon *p = 0.017*), *Faecalibacterium* (ANCOM-BC2 *log_2_FC = 1.68*, *q = 0.0046*; Wilcoxon *p = 0.019*), and *Agathobacter* (ANCOM-BC2 *log_2_FC = 2.96*, *q = 0.0007*; Wilcoxon *p < 0.001*) also showed significantly higher abundance in the UK diet before than during the Arctic diet.

In contrast, *Bacteroides* was significantly more abundant during the Arctic diet compared to the UK diet before (ANCOM-BC2 *log_2_FC = 0.446*; Wilcoxon *p = 0.04*), alongside *Alistipes* (ANCOM-BC2 *log_2_FC = 1.91*, *q = 0.0099*; Wilcoxon *p = 0.019*), *Lachnoclostridium* (ANCOM-BC2 *log_2_FC = 0.199*; Wilcoxon *p = 0.007*), *Intestinimonas* (ANCOM-BC2 *log_2_FC = 2.55*, *q = 0.0017*; Wilcoxon *p = 0.007*), and *Desulfovibrio* (ANCOM-BC2 *log_2_FC = 1.11*, *q = 0.009*; Wilcoxon *p = 0.02*).

#### 3.4.1 Taxa associated with animal- and fiber-rich dietary patterns

**Figure 6:**
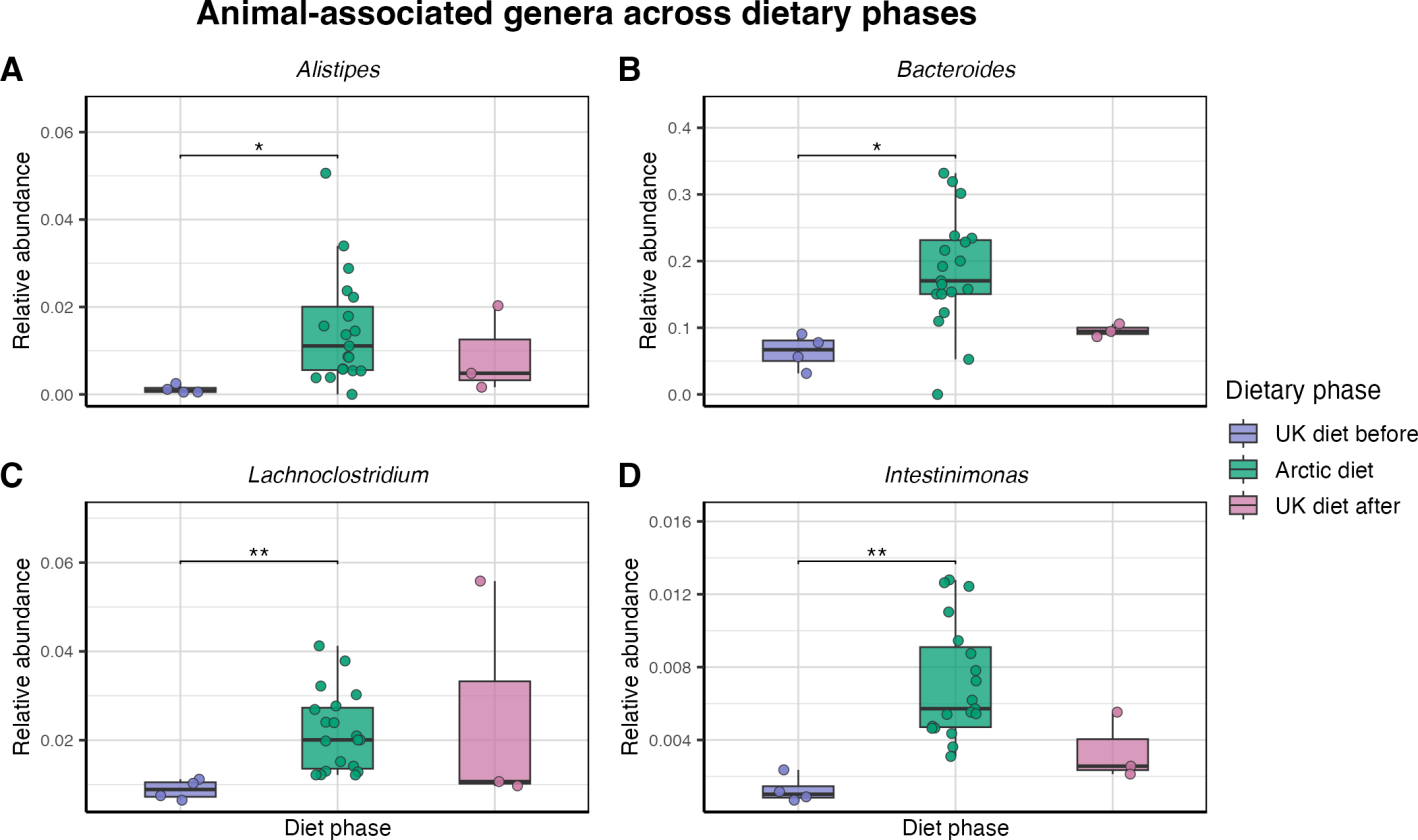
Genera associated with protein- and fat-rich dietary patterns across dietary phaess. Boxplots showing the relative abundance of selected genera across 26 fecal samples from three dietary phases: UK diet before (purple, n = 4), Arctic diet (green, n = 19), and UK diet after (pink, n = 3). Points represent individual fecal samples. Four genera were assessed: **(A)** *Alistipes*, **(B)** *Bacteroides*, **(C)** *Intestinimonas*, and **(D)** *Lachnoclostridium*. Boxes represent the interquartile range, and horizontal lines represent the median. Significance was assessed using pairwise Wilcoxon rank-sum tests with Benjamini–Hochberg correction within each genus; * indicates statistical significance. Each panel uses an independent y-axis scale.

To visualize these compositional patterns, selected genera commonly associated with animal- and fiber-rich dietary patterns were examined across the three dietary phases. Protein- and fat-associated genera (*Alistipes*, *Bacteroides*, *Lachnoclostridium*, and *Intestinimonas*) (**Figures 6A–D**) showed higher relative abundance during the Arctic diet. The increases were statistically significant for *Alistipes* (*p = 0.019*), *Bacteroides* (*p = 0.04*), *Intestinimonas* (*p = 0.007*), and *Lachnoclostridium* (*p = 0.007*) compared with the UK diet before. None of these taxa differed significantly between the Arctic and UK diet after (all adjusted p-values > 0.05).

**Figure 7:**
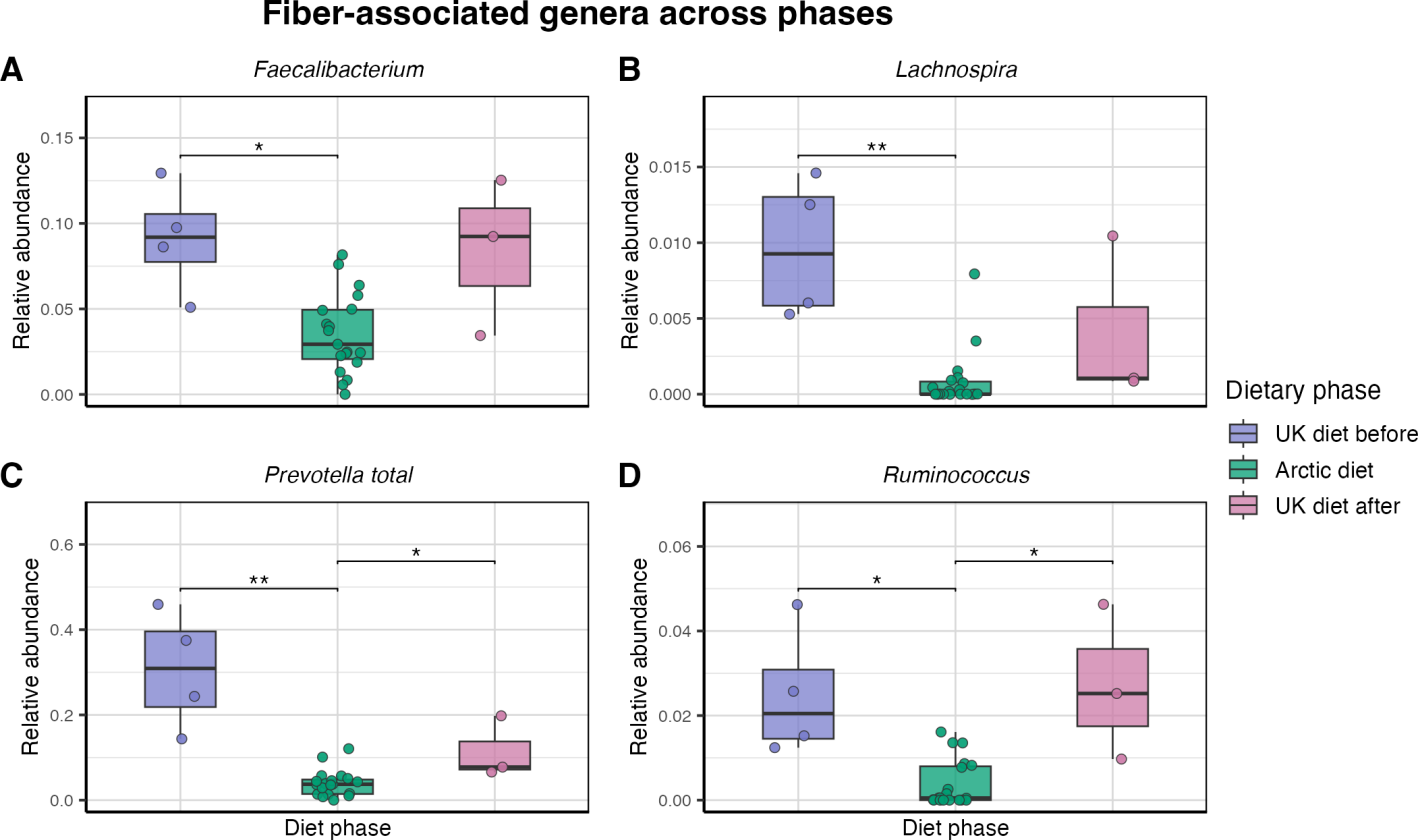
Genera associated with fiber-rich dietary patterns across dietary phases. Boxplots showing the relative abundance of selected genera across 26 fecal samples from three dietary phases: UK diet before (purple, n = 4), Arctic diet (green, n = 19), and UK diet after (pink, n = 3). Points represent individual fecal samples. Four genera were assessed: **(A)** *Faecalibacterium*, **(B)** *Lachnospira*, **(C)** *Prevotella* total summed across clades, and **(D)** *Ruminococcus*. Boxes represent the interquartile range, and horizontal lines represent the median. Significance was assessed using pairwise Wilcoxon rank-sum tests with Benjamini–Hochberg correction within each genus; * indicates statistical significance. Each panel uses an independent y-axis scale.

In contrast, fiber-associated genera (*Prevotella* (summed across clades), *Ruminococcus*, *Lachnospira*, and *Faecalibacterium*) (**Figures 7A–D**) were significantly reduced during the Arctic diet compared with the UK diet before *Faecalibacterium* (*p = 0.019*), *Lachnospira* (*p = 0.008*), *Ruminococcus* (*p = 0.017*), and *Prevotella* (p = 0.0071). Between the Arctic diet and the UK diet after, there was a significant difference in *Ruminococcus* (*p = 0.022*) and *Prevotella* (*p = 0.032*).

Given its high abundance and ecological relevance, the genus *Prevotella* was further resolved into its SILVA clades (**Supplementary Figure 4**). *Prevotella 7* showed a non-significant trend toward increasing abundance after dietary reversion to the UK diet after (*p = 0.065*). *Prevotella* 9 showed a significant decrease from the UK diet before to the Arctic diet (*p = 0.001*) and remained low after returning to the UK diet after (*p = 0.078*). The last *Prevotella* clade (unnumbered) showed no significant difference.

Complete Wilcoxon statistics for all pairwise comparisons reported here are provided in **Supplementary Table 4**.

### 3.5 Microbial composition of the ammassak gut

To characterize microbial composition of the ammassak gut, the mean relative abundance and standard deviation (SD) of the five most dominant phyla and genera were calculated.

**Figure 8:**
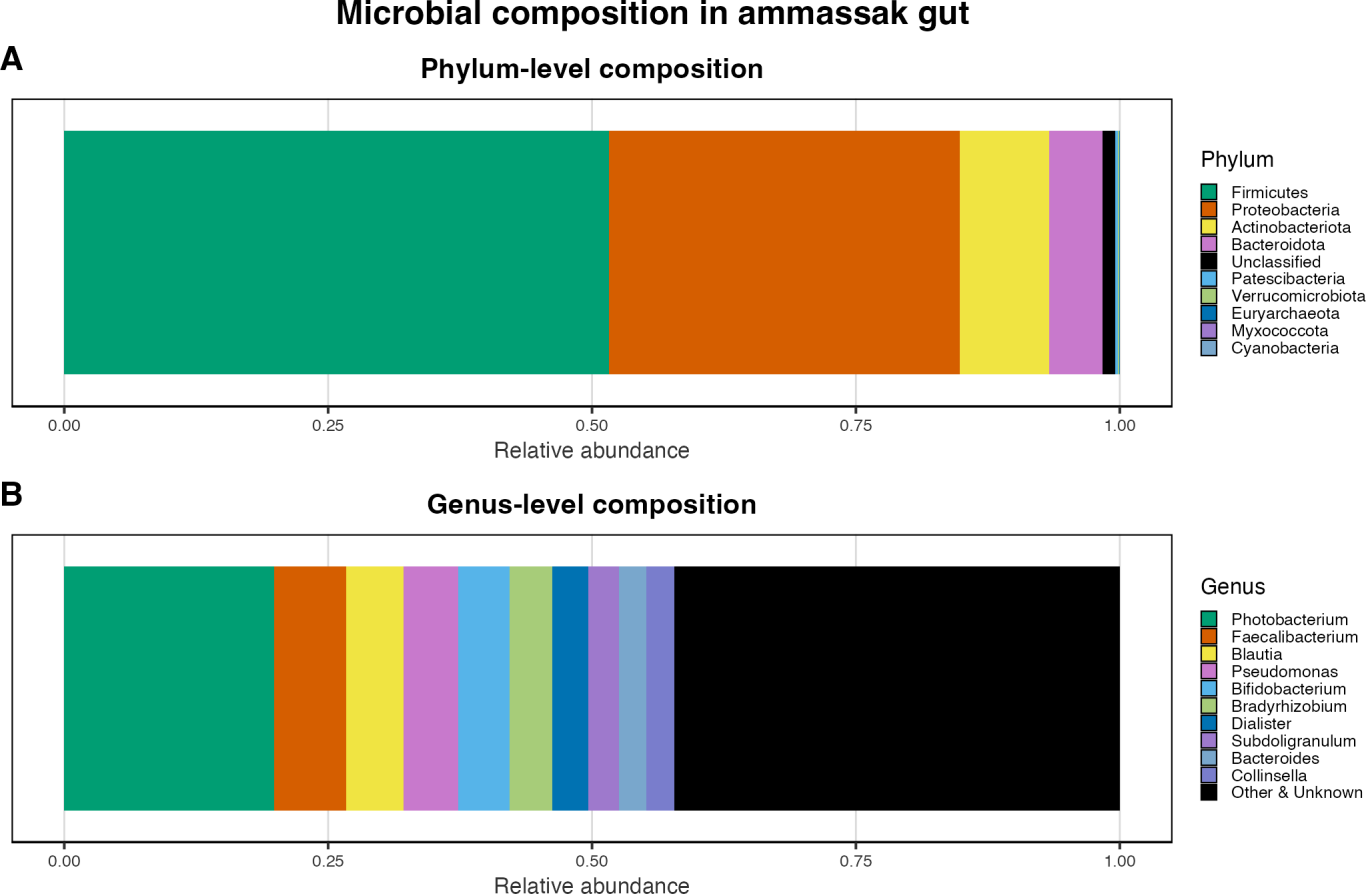
Relative abundance of dominant bacterial taxa in ammassak gut samples. **(A)** Mean relative abundance of dominant bacterial phyla in the ammassak gut microbiota (n = 11). **(B)** Mean relative abundance of the 10 most abundant bacterial genera in the ammassak gut microbiota. Taxa not among the top 10 most abundant genera are grouped as “Other & Unknown”.

At the phylum level, the microbial community was dominated by *Firmicutes* (51.61% ± 23.64%), followed by *Proteobacteria* (33.2% ± 30.8%), *Actinobacteriota* (8.52% ± 5.1%), *Bacteroidota* (5.01% ± 3.35%), and some unknown phyla (1.24% ± 1.03%) (**Figure 8A**). At the genus level, the most abundant taxa were *Photobacterium* (19.9% ± 33.81%), *Faecalibacterium* (6.78% ± 4.04%), *Blautia* (5.44% ± 3.3%), and *Pseudomonas (5.22%* ± 11.5%), *Bifidobacterium* (4.84% ± 3.27%) (**Figure 8B**).

#### 3.5.1 Introduced genera shared with the ammassak gut

To examine overlap between genera detected in the ammassak gut and genera newly detected in the participant’s gut microbiota during the Arctic diet, we compared the genera present in the ammassak gut with those detected in the participant’s gut microbiota across all dietary phases (**Supplementary Figure 3**). Four genera were identified as absent from the participant’s gut microbiota during the UK diet before and pre-ammassak but exceeded a relative abundance threshold of 0.1% during or after the period of ammassak consumption. The genera *Slackia* and *Lachnospiraceae UCG-010* were first detected during the Arctic diet and remained present in the post-ammassak. *Photobacterium* and *Turicibacter* were detected after the ammassak intervention during the post-ammassak. These four genera were also present in the ammassak gut microbiota dataset.

## 4 Discussion

### 4.1 Arctic diet and the gut microbiota

Diet is one of the most influential environmental factors shaping the gut microbiota (Turnbaugh et al., 2009; David et al., 2014). Non-Western diets are generally associated with greater microbial diversity and the presence of microbial taxa rarely observed in Westernized counterparts (De Filippo et al., 2010; Conlon and Bird, 2015; Martínez et al., 2015). Western diets, characterized by an abundance of ultra-processed foods and saturated fats, have been consistently linked to reduced microbial diversity and a decrease in beneficial commensals (De Filippo et al., 2010; Sonnenburg and Sonnenburg, 2014). These patterns are linked to dysbiosis, including decreased short-chain fatty acid (SCFA) production and a shift toward pro-inflammatory microbial profiles. Such changes form the basis of the “disappearing microbiota” hypothesis, which suggests that industrialized lifestyles are gradually eroding the human gut microbiota, with potential long-term consequences for health (Blaser and Falkow, 2009; Sonnenburg and Sonnenburg, 2019; Leeming et al., 2021). Although the microbiota-enriching potential of plant-based, non-Western diets has been well characterized, animal-based, non-Western diets remain underrepresented in microbiome studies. Understanding how such animal-based diets influence gut microbial dynamics is particularly relevant in the Arctic. Previous studies’ characterization of Indigenous Inuit gut microbiomes (Dubois et al., 2017; Girard et al., 2017) relied on cross-sectional comparisons of individuals consuming mixed Indigenous-Western and Western diets, making it difficult to distinguish microbial shifts specifically associated with Indigenous Arctic dietary practices.

Because the study included only one participant, the findings reflect an individual response and cannot exclude the influence of baseline microbiota composition, host physiology, or other individual-specific factors. In addition, because the Arctic diet transition overlapped significantly with a prolonged qajaq expedition involving sustained physical activity, the design does not allow the independent effect of diet and physical activity to be separated. Diet is widely recognized as a major determinant of gut microbial structure, and controlled human studies have shown that marked short-term shifts can rapidly alter gut microbial community composition (David et al., 2014; Rinninella et al., 2023). In contrast, the effects of physical activity on gut microbial diversity and composition in humans remain heterogeneous and are frequently difficult to disentangle from dietary and other host factors (Ortiz-Alvarez et al., 2020; Dorelli et al., 2021; Cullen et al., 2023). Although the independent effects of diet and sustained physical activity during the qajaq expedition cannot be separated here, several of the taxonomic shifts observed during the Arctic diet were consistent with the change in dietary substrate, particularly the transition from a fiber-containing Western diet to a strictly Indigenous Arctic animal-based diet.

### 4.2 Microbial diversity and community structure

Here, we observed that the Arctic diet was associated with changes in the human gut microbial community structure, without a reduction in alpha diversity, and that these changes were only partially reversed after returning to the Western diet. Although the study involved a single participant, the results showed a distinct separation between Western and Arctic diets within 7 days. This observation aligns with previous studies, showing that even short-term dietary interventions can induce measurable changes in microbial community structure (David et al., 2014; Singh et al., 2017).

In contrast to the common notion that plant-based diets are crucial for gut microbial diversity, alpha diversity (**Figures 2A–D**) remained steady during the Arctic diet. Although these differences were not statistically significant, the observed patterns suggest that a transition to this Arctic dietary context was not associated with a reduction in alpha diversity. This adds nuance to previous findings, in which diets rich in animal products in a Western context were associated with reduced alpha diversity in short-term interventions (David et al., 2014), and in which plant-based, high-fiber diets were found to support higher microbial richness (De Filippo et al., 2010). Alpha diversity also appeared to remain elevated during the short follow-up after returning to the Western diet.

The microbial composition of the gut microbiota changed significantly during the Arctic diet. Beta diversity analysis based on Bray-Curtis dissimilarity (**Figure 3**) revealed a statistically significant shift in microbial community composition during the Arctic diet compared to both UK diet phases. Notably, the earliest Arctic diet samples were collected before the qajaq expedition began on April 26th, and these samples were already separated from the UK diet before in the NMDS ordination. This pattern is consistent with a contribution of the dietary transition to the initial microbial restructuring, although the changes for the later part of this period cannot be disentangled from the overlap with the physical activity associated with the qajaq expedition. Within the brief period assessed, the transition back to a Western diet only partially reversed the changes in the gut microbiota. While some microbial features returned to their pre-intervention state, other taxa introduced during the Arctic diet were no longer detectable once the participant resumed a Western diet, indicating that the microbial shifts during the intervention were not immediately reversed upon returning to a Western diet. This might be explained by ecological resilience or niche exclusion (Sonnenburg et al., 2016; Valdes et al., 2018; Zmora et al., 2018). Our findings add to a growing body of evidence suggesting that even short-term dietary interventions can induce microbial restructuring that is not immediately reversible, particularly when new taxa occupy unique niches or are reinforced by sustained dietary exposure (Leeming et al., 2019; Wastyk et al., 2021). Rampelli et al. (2025) reported recently that consumption of wild foods induced gut microbiota alterations that were only partially reversed after the intervention. The pairwise PERMANOVA comparing the UK diet before and after indicated a significant difference between them, suggesting that the Arctic diet phase induced compositional changes that persisted during the post-intervention period. At the genus level, the ANCOM-BC2 and Wilcoxon tests revealed significant decreases in the *Prevotella* 9 clade, *Faecalibacterium*, *Ruminococcus*, and *Lachnospira* during the Arctic diet, and an increase in *Bacteroides*, *Alistipes*, *Intestinimonas*, and *Lachnoclostridium*. Although several of these taxa tended to return toward baseline after the diet reversal, none fully restored within the observed timeframe. In particular, *Prevotella* 9 remained depleted after the intervention, while *Prevotella* 7 showed a modest increase; however, this increase was not statistically significant. The comparison of timescales highlights an important asymmetry: the gut microbiota shifted rapidly from the Western baseline to a new configuration within just one week of adopting the Arctic diet, yet recovery toward this baseline proceeded more slowly and remained incomplete after a month. This asymmetry might reflect differences in microbial exposure between diets. The Arctic diet, rich in raw and fermented foods, has the potential to introduce a substantial influx of environmental and foodborne microbes, accelerating community turnover. In contrast, the return to the Western diet, which is largely microbially depleted due to industrial processing (Marco et al., 2017; Sonnenburg and Sonnenburg, 2019), offers limited microbial input (David et al., 2014; Leeming et al., 2019; Wastyk et al., 2021).

### 4.3 Compositional shift and microbial turnover during the Arctic diet

At the phylum level, the *Firmicutes*/*Bacteroidota* (F/B) ratio increased from 1.31 before dietary intervention to 2.12 during the Arctic diet and remained elevated at 2.38 after returning to the Western diet. High F/B ratios are often associated with Western, high-fat, low-fiber diets (David et al., 2014; Magne et al., 2020), whereas non-Western, plant-based diets typically display lower ratios and a higher abundance of *Bacteroidota* taxa (De Filippo et al., 2010; Martínez et al., 2015). A reorganization within the phylum *Bacteroidota* was observed, with *Bacteroides*, a genus linked to protein and fat metabolism (Wu et al., 2011), increasing during the Arctic diet, while the *Prevotella* 9, which is typically dominant under carbohydrate- and fiber-rich conditions (De Filippo et al., 2010), declined sharply after transition to the Arctic diet. These genus-level shifts are consistent with patterns described in a recent review by Daunizeau et al. (2025), which compared multiple Indigenous peoples’ gut microbiomes and their more Westernized counterparts. Across most of the Indigenous populations reported in that review, *Prevotella* was enriched, and *Bacteroides* was reduced relative to Westernized groups; however, Inuit were a notable exception. Among Inuit, individuals who reported more frequent consumption of Indigenous Arctic foods exhibited lower *Prevotella* abundance and higher *Bacteroides* abundance than those with more Westernized diets. Daunizeau et al. (2025) also reported an elevated *Firmicutes*/*Bacteroidota* ratio among Inuit with more frequent intake of Indigenous Arctic foods. The direction of change observed in our study, a decline in certain *Prevotella* clades, enriched *Bacteroides*, and an elevated F/B ratio during the Arctic diet, reproduced these microbial patterns reported here.

Taxonomic composition profiles (**Figures 4A, B**) supported these genus-level trends, showing a clear shift in the relative abundance of dominant taxa as dietary intake changed. Beta-diversity analysis based on Bray-Curtis dissimilarity (**Figure 3**) corroborated this pattern, revealing a change in the overall microbial community structure between diet phases and partial recovery following reversion to the Western diet. Several genera typically associated with fiber-rich diets, including *Prevotella 9*, *Bifidobacterium*, *Lachnospira*, and *Ruminococcus*, were statistically more abundant under the UK diet (**Figures 7A–D**), likely reflecting a higher intake of fermentable carbohydrates relative to the Arctic diet.

In contrast, genera commonly linked to protein- and fat-based diets, including *Bacteroides* and *Alistipes* (David et al., 2014), *Lachnoclostridium* (Wu et al., 2022), and *Intestinimonas* (Bui et al., 2015; Rampanelli et al., 2025), were significantly enriched during the Arctic diet (**Figures 6A–D**), reflecting a microbial community better adapted to the nutrient profile of Inuit foods. Several of these taxa, particularly *Alistipes* and *Intestinimonas*, have been associated with the production of SCFA through amino-acid fermentation (Morrison and Preston, 2016; Parker et al., 2020; Zhu et al., 2021), suggesting that the Arctic diet was associated with microbial restructuring consistent with adaptation to protein- and fat-rich, low-fiber conditions. Together, these patterns reflect a community restructuring towards optimized protein and fat metabolism. The sustained elevation of the F/B ratio after return to the UK diet aligns with the ASV-overlap pattern (**Figure 4C**), which showed limited overlap between the two UK diet phases but a greater number of shared ASVs between the Arctic diet and the adjacent phases. This pattern suggest that the Arctic diet introduced new taxa, some of which remained detectable after the dietary reversion, possibly reflecting short-term ecological resilience within the restructured community, delayed recovery of taxa reduced during the broader Arctic diet period, or temporary niche occupation by taxa favored during that period. In this context, ecological resilience refers to the tendency of the restructured community to maintain its altered composition after dietary exposure ends, whereas niche occupation or exclusion may limit the immediate re-expansion of taxa that were reduced during the Arctic diet. The greater ASV overlap between the Arctic diet and the adjacent phases is consistent with the persistence of some taxa beyond the intervention itself. Previous work has shown that some microbial shifts can persist beyond short-term interventions, depending on host and ecological factors (David et al., 2014; Sonnenburg et al., 2016). The observed pattern is consistent with these ecological processes, although their relative contribution cannot be determined here due to the single-participant design and short follow-up. Longer follow-up and complementary approaches, such as metagenomics and metabolomics, will be needed to determine whether these compositional shifts are sustained and whether they translate into functional changes.

Indigenous peoples’ plant-based diets have consistently been associated with higher gut microbial richness compared to Western diets, due to the greater variety and complexity of fermentable plant fibers in non-Western diets (De Filippo et al., 2010; Sonnenburg and Sonnenburg, 2019). The Arctic diet in this study, while also non-Western, contrasts with previously considered non-Western diets, as it is primarily animal-based and low in fermentable fiber. Despite this, we did not observe a decline in microbial richness, highlighting that fermentable fiber is not the sole source of increased gut microbiota diversity. One plausible cause of increased microbial diversity in various non-Western diets is the absence of industrial food processing, a feature shared by Indigenous peoples’ plant- and animal-based diets. Minimally processed foods are more likely to retain their native microbial communities, structural complexity, and chemical diversity, all of which may contribute to the enrichment of the gut microbiota (Sonnenburg and Sonnenburg, 2019; Wang et al., 2021; Wastyk et al., 2021). Whereas plant-based, non-Western diets have been linked to higher diversity and a lower F/B ratio through fiber-driven microbial richness (De Filippo et al., 2010; Martínez et al., 2015), the Arctic diet appears to enhance diversity through other pathways. These include the availability of microbial substrates such as amino acids and lipids, exposure to environmental microbes through handling and consumption of a high number of raw and fermented animal-source foods, and practices such as gastrophagy, which involves ingesting intestinal contents and associated microbiota.

Together, these findings demonstrate that the Arctic diet was associated with a distinct restructuring of the gut microbiota, characterized by the enrichment of taxa specialized in protein and fat metabolism, and sustained community alterations even after returning to the Western diet.

### 4.4 Microbial shift and potential foodborne taxa

In this study, we assessed the consumption of dried ammassak, which is traditionally eaten whole, including the gut contents (Hauptmann et al., 2020). The differential abundance (**Figure 5A**) and presence-absence analyses (**Supplementary Figure 3**) showed that the transition to the Arctic diet reshaped the relative abundance of introduced gut taxa and was associated with the detection of several new genera previously undetected. This interpretation is supported by the Arctic diet subphase analyses, which showed no significant differences in alpha diversity between pre-ammassak, ammassak-intervention, and post-ammassak (**Supplementary Figure 1**) but significant differences in beta diversity across all three subphases. Together, these results suggest that community composition continued to restructure during the Arctic diet, while alpha diversity remained relatively stable. ANCOM-BC2 identified nine genera that increased and 19 that declined during the Arctic diet. Complementary presence-based analyses identified 27 genera that appeared during this period and seven genera that disappeared. Notably, *Slackia* and *Lachnospiraceae UCG-010* appeared during the ammassak consumption period, coinciding with the ingestion of whole, fermented fish and the practice of gastrophagy. Furthermore, *Photobacterium* and *Turicibacter* were first detected in the subsequent post-ammassak period. The overlap between these taxa and those identified in the ammassak gut microbiota is consistent with potential dietary exposure during ammassak consumption, in which gastrophagy occurred, although this it does not indicate stable colonization. However, because the Arctic subphases also reflect temporal progression during the expedition, these temporal patterns cannot be attributed to ammassak consumption alone. Nevertheless, the timing of the appearance of several genera during or after the ammassak-intervention, together with their overlap with taxa detected in the ammassak gut microbiota, makes ammassak a plausible contributor to the observed microbial turnover.

Foods themselves carry live microbes; ingestion therefore represents a microbial exposure that can result in transient detection in stool even without stable colonization (Lang et al., 2014). The timing and identity of *Photobacterium* are consistent with potential dietary introduction. *Photobacterium* was a highly dominant genus in the intestinal microbiota of the ammassak (**Figure 8B**) and is a marine-associated genus reported in dried and traditionally processed fish, including ammassak (Hauptmann et al., 2020; Bahrndorff et al., 2022). Its detection only after the ammassak consumption suggests a delayed or transient appearance, possibly reflecting short-term exposure through the diet or other environmental inputs rather than stable colonization. Although *Photobacterium* was not statistically significant in the ANCOM-BC2 analysis, its temporal pattern remains biologically noteworthy when considered alongside presence-absence dynamics, supporting the notion of transient microbial exposure through foods (Lang et al., 2014).

These findings are relevant in the light of growing evidence that fermented and unprocessed foods can influence the gut microbiota through multiple mechanisms beyond persistent colonization. In addition to direct microbial exposure, food-associated taxa may contribute to short-term ecological interactions, metabolic cross-feeding, or modulation of the gut environment through bioactive metabolites (Wastyk et al., 2021).

To further explore the microbial dynamics associated with the Arctic diet, we examined the genera that appeared only after the dietary shift. Of the 47 introduced genera, 27 were consistently detected (**Supplementary Figure 3**, **Supplementary Table 3**), defined as being present in at least two samples within one or more of the Arctic diet subphases and at a relative abundance of at least 0.1%. This more restricted set suggests that at least some newly detected taxa were not merely sporadic observations but appeared repeatedly during the Arctic diet. These taxa may reflect potential food-associated microbial exposure, including from ammassak, or broader ecological restructuring of the gut microbiota under altered nutrient conditions (David et al., 2014; Sonnenburg and Sonnenburg, 2019; Hauptmann et al., 2020). Together, these findings suggest that the Arctic diet was associated not only with shifts in the relative abundance of existing taxa but also with the repeated detection of taxa not observed during the preceding Western diet period.

### 4.5 Decline of fiber-associated and beneficial taxa

Among the genera that declined during the Arctic diet, *Bifidobacterium* is notable for its well-established roles as a beneficial commensal and as a widely recognized probiotic. Members of this genus contribute to gut health through carbohydrate fermentation, acetate production, modulation of immune function, and the competitive exclusion of pathogens (O’Callaghan and van Sinderen, 2016; Ruiz et al., 2017). Its decline likely reflects reduced availability of fermentable fibers during the intervention, a crucial substrate for *Bifidobacterium*. Butyrate-producing genera such as *Faecalibacterium* and *Agathobacter* also decreased during the Arctic diet, likely due to the limited availability of fermentable fibers required for their growth (Louis and Flint, 2017). The decline in these genera may indicate reduced abundance of taxa commonly associated with SCFA production by the gut microbiota. It is important to note that protein fermentation also produces SCFA, and that dried ammassak has been shown, in some cases, to contain significant amounts of lactic acid and propionic acid-producing bacteria (Hauptmann et al., 2020).

The Arctic diet was also associated with the expansion of other taxa that have potential beneficial metabolic roles. For instance, genera such as *Lachnoclostridium*, *Intestinimonas*, and *Alistipes*, although not characterized as probiotics, have been linked to metabolic flexibility and SCFA production under low-fiber conditions (Parker et al., 2020; Wang et al., 2021; Zhu et al., 2021), suggesting that microbial communities shaped by non-Western animal-based diets may differ compositionally yet still retain functional capacity through alternative metabolic pathways.

### 4.6 Implications for microbial resilience and traditional food systems

The practice of gastrophagy, consuming animals’ intestines and their contents, as occurs when eating dried ammassak, represents a distinctive route of microbial exposure. Unlike most industrial food systems that minimize microbial contact, this practice may introduce live, gut-associated microbes into the human digestive tract. Although the present study cannot establish causality, the transient detection of foodborne taxa, such as *Photobacterium*, supports the idea that Indigenous Arctic foods may contribute to microbial input during dietary transition. Taken together, these findings highlight that traditional foods may shape the gut microbiota through nutrients and microbial exposure that are rarely encountered in ultra-processed diets.

### 4.7 Limitations

An important limitation of this study is that the Arctic diet period overlapped substantially with a qajaq expedition that involved sustained high physical activity and expedition-related environmental changes. As a result, the observed shift in the gut microbiota cannot be attributed to diet alone. In this single-participant design, these concurrent exposures could not be meaningfully disentangled, and the Arctic diet period should therefore be interpreted as a combined dietary and lifestyle exposure period rather than a diet-only intervention. Accordingly, the findings are best viewed as hypothesis-generating and should be validated in larger studies in which diet, host factors, physical activity, and environmental context can be more clearly separated.

## 5 Conclusion

This study shows that transitioning from a Western to an Indigenous Arctic dietary context was associated with marked changes in the human gut microbiota, even within a short time frame and under real-life conditions. The results highlight the gut community’s responsiveness to dietary change and its capacity to adapt to the nutrient composition of animal-based foods.

A transient appearance of potentially foodborne taxa, such as *Photobacterium*, suggests that microbial exposure may occur through consumption of traditional foods, including ammassak, and through gastrophagy. Despite the low fiber content of the Arctic diet, microbial diversity remained stable, underscoring that fermentable fiber is not the sole driver of gut microbial richness. The gut microbial community during the Arctic diet was consistent with adaptation to the nutrient profile of the Indigenous Arctic diet through an increased *Firmicutes*/*Bacteroides* (F/B) ratio, as well as a microbiota that shifted toward specialization in protein and fat metabolism, with an increased relative abundance of *Bacteroides*, *Alistipes*, and *Intestinimonas*, and a decline in fiber-associated genera, such as *Prevotella*.

Overall, the findings highlight the adaptability of the human gut microbiota to substantial dietary change. Although based on a single participant, the high-resolution temporal data provide a rare within-subject view of how Indigenous Arctic food practices interact with gut microbial ecology. Future research should examine these patterns in larger cohorts and integrate metagenomics, metabolomics, and dietary modeling to clarify how the broader transition from a fiber-containing diet to a strictly Indigenous Arctic animal-based diet, as well as exposure to traditional foods such as ammassak, shapes gut microbial composition and function.

## Data Availability

The data presented in the study are deposited in the NCBI repository, accession number PRJNA1431428.
