# Supplementary material for: Dietary transition to an Indigenous Greenlandic diet induces instant shifts in gut microbiota composition – a pilot intervention study
Source: Front Microbiomes. 2026 May 21;5:1832705. doi: 10.3389/frmbi.2026.1832705 (PMC13234626; doi:10.3389/frmbi.2026.1832705)
Supplement: Supplementary file 1 [file Image1.pdf]

Supplementary Figure S1

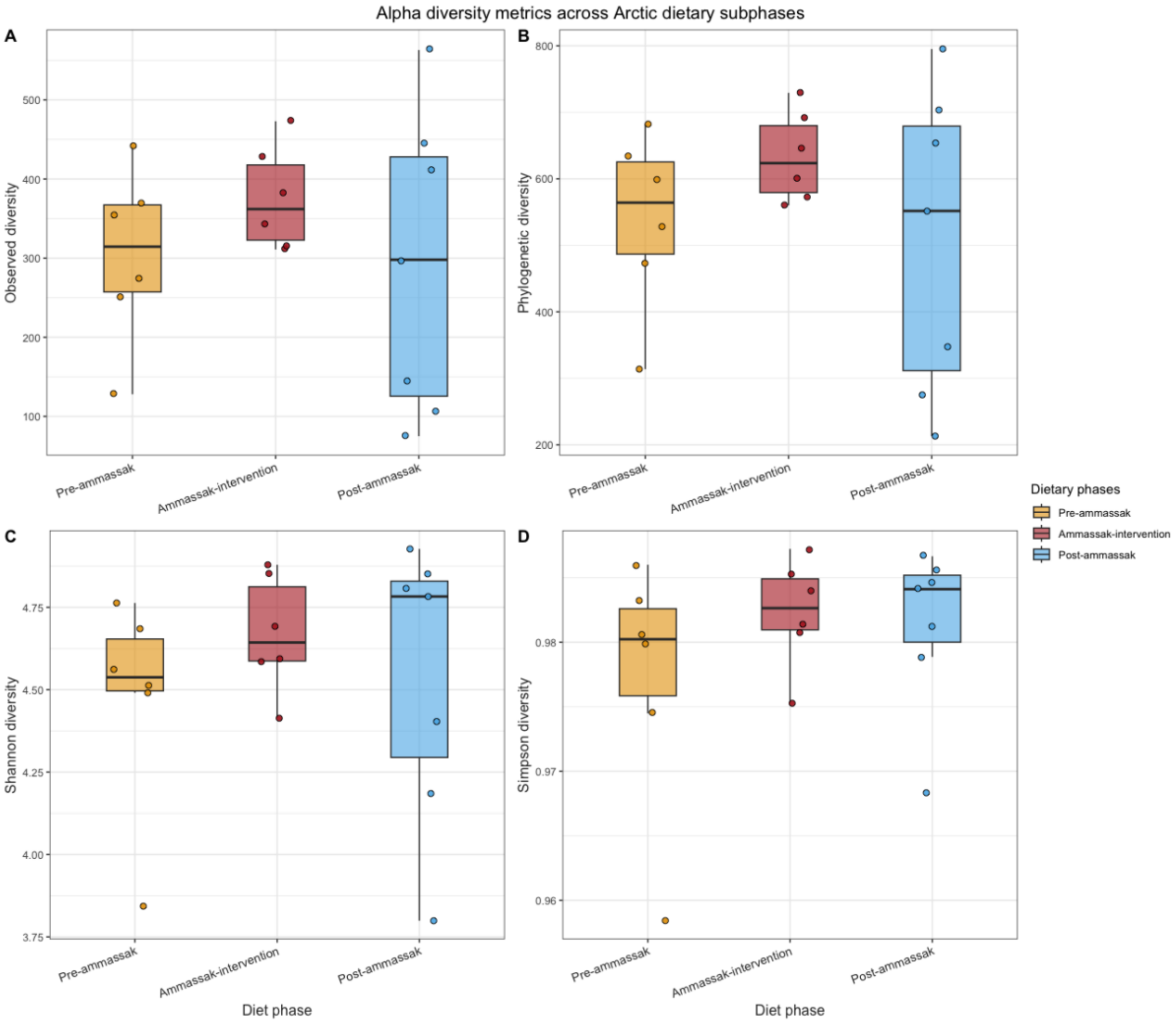

Supplementary Figure S1. Alpha diversity of the human gut microbiota across Arctic dietary subphases.

Boxplot showing within-sample microbial diversity across 19 fecal samples from three Arctic dietary subphases: pre-ammassak (orange, n = 6), ammassak-intervention (red, n = 6), and post-ammassak (blue, n = 7). Points represent individual fecal samples. Four alpha diversity metrics were assessed: (**A**) Observed richness, (**B**) Phylogenetic diversity (Faith's PD), (**C**) Shannon index, and (**D**) Simpson index. No significant differences were detected (all $p < 0.05$).
