# Supplementary material for: Dietary transition to an Indigenous Greenlandic diet induces instant shifts in gut microbiota composition – a pilot intervention study
Source: Front Microbiomes. 2026 May 21;5:1832705. doi: 10.3389/frmbi.2026.1832705 (PMC13234626; doi:10.3389/frmbi.2026.1832705)
Supplement: Supplementary file 2 [file Image2.pdf]

Supplementary Figure S2

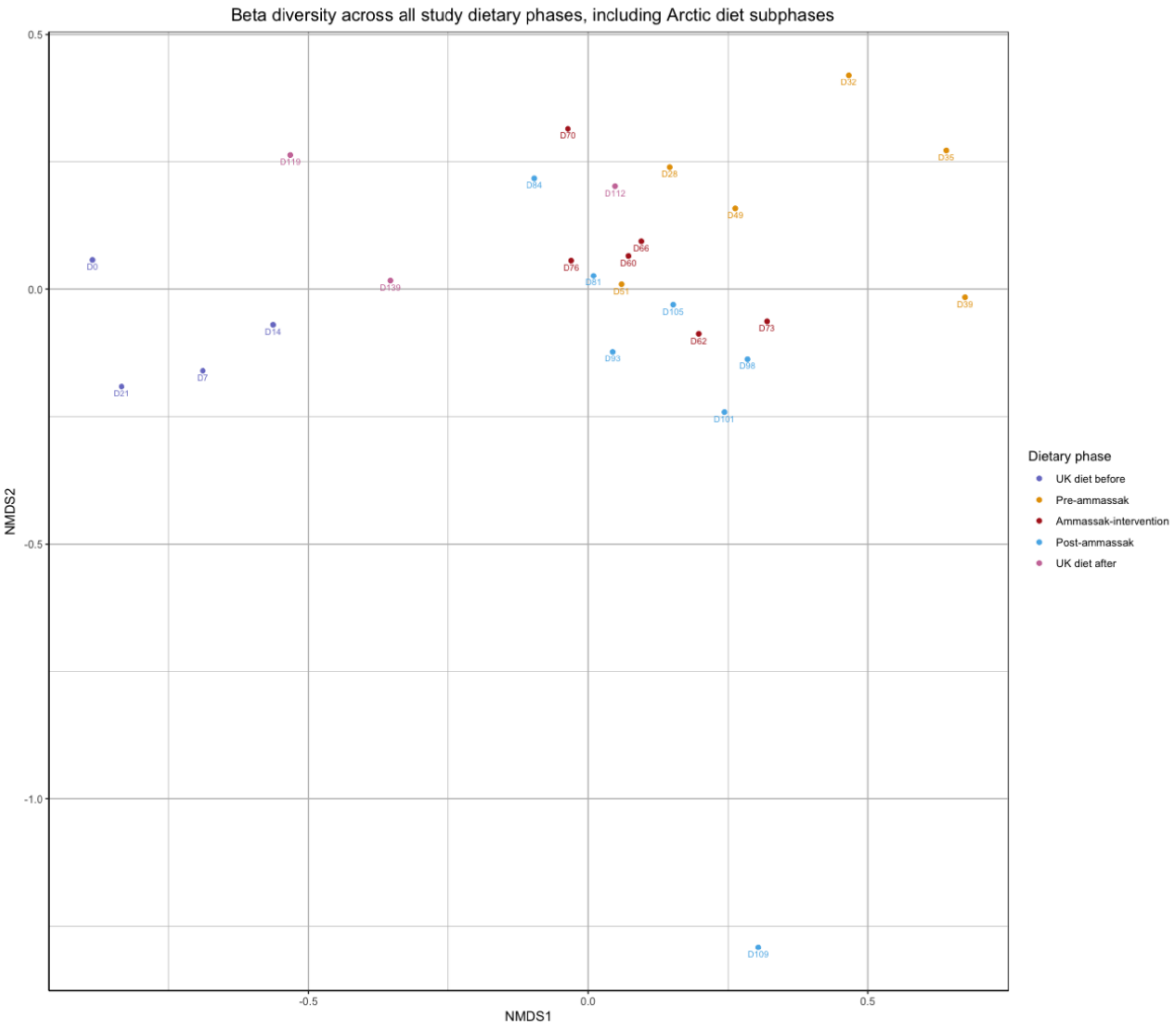

Supplementary Figure S2. Beta diversity of the human gut microbiota across dietary phases with Arctic subphase resolution (Bray-Curtis, NMDS).

Non-metric multidimensional scaling (NMDS) based on Bray-Curtis dissimilarity illustrating gut microbial community composition across 26 fecal samples from five study phases: UK diet before (purple, n = 4), pre-ammassak (orange, n = 6), ammassak-intervention (red, n = 6), post-ammassak (blue, n = 7), and UK diet after (pink, n = 3). Each point represents the microbial composition of a sample and is labeled with the number of days from the start of the study. Each number represents the day the sample was taken, with D0 (purple) being the first sample day and D139 (pink) being the last sample date.
