# Supplementary material for: Dietary transition to an Indigenous Greenlandic diet induces instant shifts in gut microbiota composition – a pilot intervention study
Source: Front Microbiomes. 2026 May 21;5:1832705. doi: 10.3389/frmbi.2026.1832705 (PMC13234626; doi:10.3389/frmbi.2026.1832705)
Supplement: Supplementary file 3 [file Image3.pdf]

Supplementary Figure S3

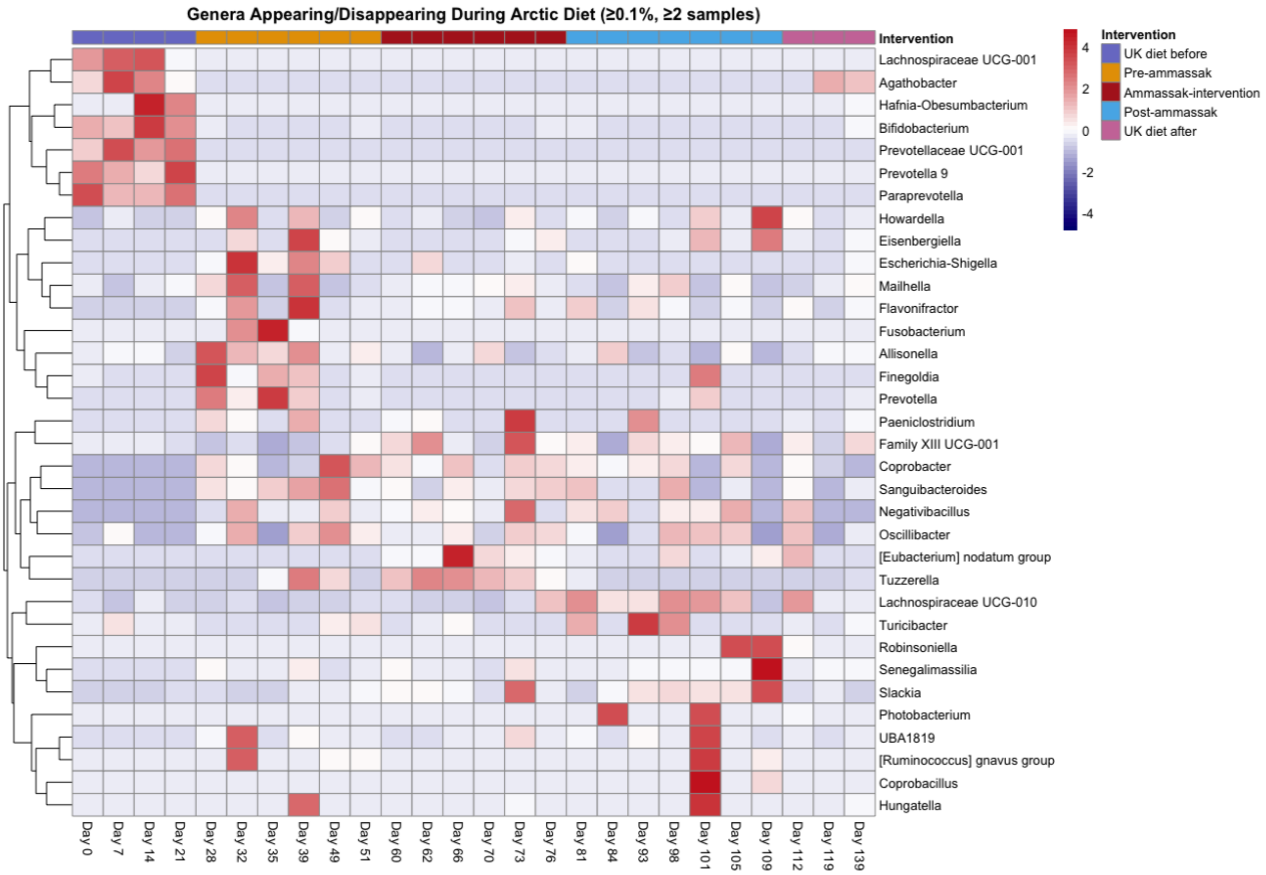


Supplementary Figure S3. Genera appearing or disappearing during the Arctic diet phase. Heatmap showing selected genera that appeared or disappeared during the Arctic diet phase, defined as genera reaching at least 0.1% relative abundance in one or more samples. Columns represent 26 fecal samples ordered by dietary phase: UK diet before (purple, n = 4), pre-ammassak (orange, n = 6), ammassak-intervention (red, n = 6), post-ammassak (blue, n = 7), UK diet after (pink, n = 3). The annotation above the heatmap indicates dietary phases and Arctic diet subphases. Color intensity represents centered and scaled relative abundance (Z-scores), with red indicating higher and blue indicating lower abundance relative to the mean abundance of each genus across samples.
