# Supplementary material for: Dietary transition to an Indigenous Greenlandic diet induces instant shifts in gut microbiota composition – a pilot intervention study
Source: Front Microbiomes. 2026 May 21;5:1832705. doi: 10.3389/frmbi.2026.1832705 (PMC13234626; doi:10.3389/frmbi.2026.1832705)
Supplement: Supplementary file 4 [file Image4.pdf]

Supplementary Figure S4

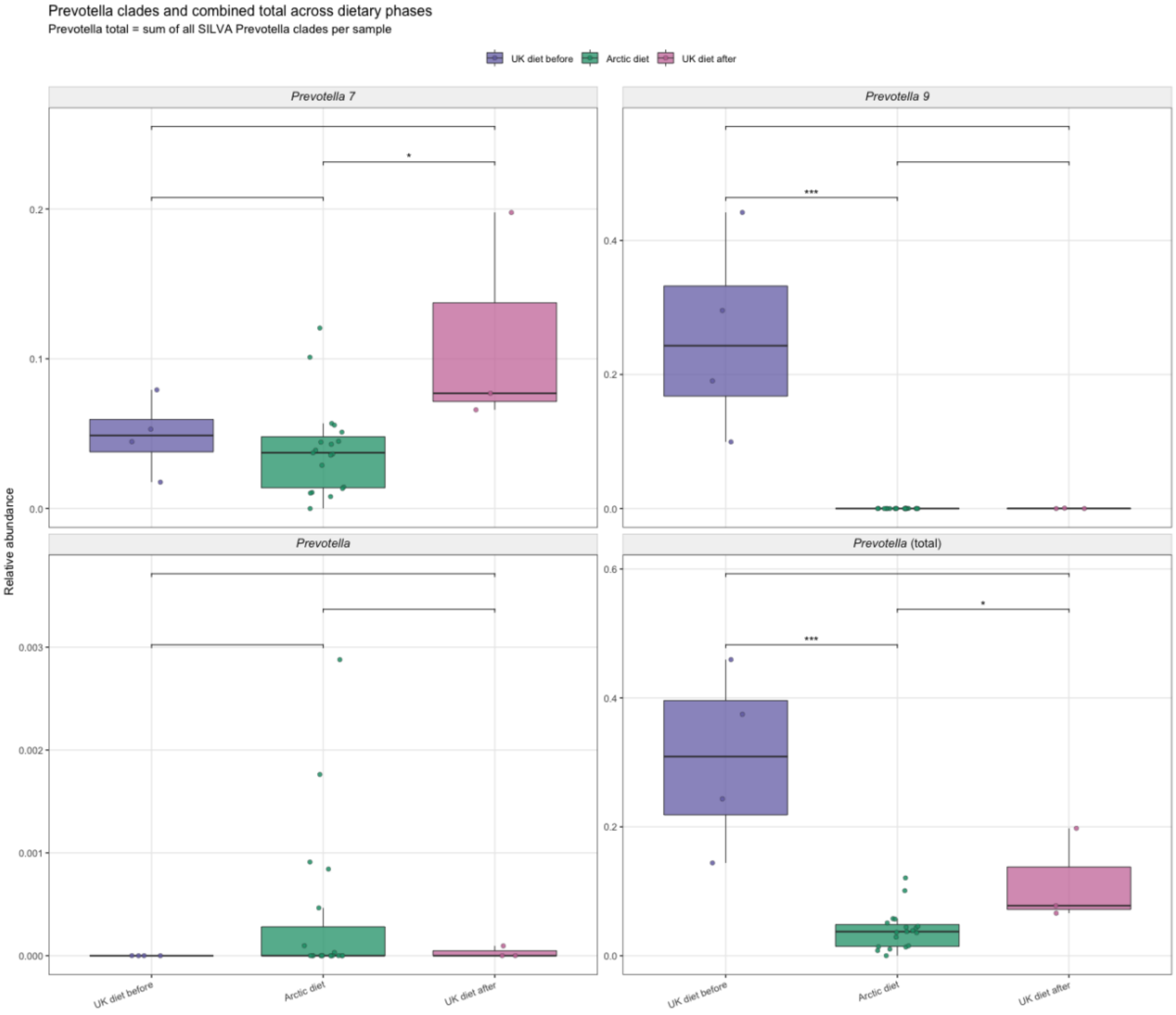

Supplementary Figure S4. Relative abundance of Prevotella clades across dietary phases.

Relative abundance of Prevotella clades (Prevotella 7, Prevotella 9, and uncultured Prevotella) and their abundance across the three dietary phases: UK diet before (n = 4), Arctic diet (n = 19), and UK diet after (n = 3). Boxes represent the interquartile range, horizontal lines the median, and points individual fecal samples. Significance was assessed with pairwise Wilcoxon rank-sum tests with Benjamini-Hochberg correction; * indicates significant differences. Each facet uses its own y-axis scale.
