# Supplementary material for: Dietary transition to an Indigenous Greenlandic diet induces instant shifts in gut microbiota composition – a pilot intervention study
Source: Front Microbiomes. 2026 May 21;5:1832705. doi: 10.3389/frmbi.2026.1832705 (PMC13234626; doi:10.3389/frmbi.2026.1832705)
Supplement: Supplementary file 5 [file Table1.pdf]

Supplementary Table S1. Example days from the participant's self-reported typical UK-based Western diet before and after the qajaq expedition.

Day	Foods and beverages reported	General dietary features illustrated
Day 1	1 coffee; 100 g cooked eggs; 50 g sourdough bread with 10 g butter; 120 g avocado; 250 ml raw milk; 60 g cooked ham; 300 g bolognese (beef, tomatoes, onion, garlic); 25 g 80% chocolate	Mixed omnivorous diet including eggs, bread, dairy, meat, and plant foods.
Day 2	2 coffees; 50 g cooked lamb liver; 50 g cooked egg; 100 g cooked eggs; 50 g cooked chicken thigh; 250 g cooked aubergine with miso; 50 g rice; 100 g pasteurised cheese; 25 g 80% chocolate	Mixed omnivorous diet including offal, poultry, rice, dairy, and plant foods
Day 3	1 coffee; 100 g cooked eggs; 50 g sourdough bread with 10 g butter; 50 g pasteurized cheese; 50 g sourdough bread; 120 g tinned sardines; 15 g rocket; 15 g homemade dressing (oil, vinegar, honey, herbs)	Mixed omnivorous diet including bread, dairy, fish, eggs, and plant foods
Day 4	1 coffee; 150 ml kefir; 100 g cooked eggs; 40 g pasteurised cheese; 50 g sourdough bread with 10 g butter; 60 g cooked beef liver; 40 g beef kidneys; 250 g pizza (50% bread, 15% tomato sauce, 15% cheese, 20% salami and chicken); 300 ml hot chocolate; 25 g 80% chocolate	Mixed omnivorous diet including fermented dairy, bread, offal, pizza, and other commercially prepared foods

The participant reported that his diet before and after the qajaq expedition was broadly similar and representative of his typical UK-based Western diet.
