# Supplementary material for: Dietary transition to an Indigenous Greenlandic diet induces instant shifts in gut microbiota composition – a pilot intervention study
Source: Front Microbiomes. 2026 May 21;5:1832705. doi: 10.3389/frmbi.2026.1832705 (PMC13234626; doi:10.3389/frmbi.2026.1832705)
Supplement: Supplementary file 7 [file Table3.pdf]

**Supplementary Table S3. Genera appearing or disappearing during the Arctic diet phase under permissive and strict calling rules.**

| Genus | Appear (no thr, ≥1) | Appear (0.1% & ≥2) | Disappear (no thr, ≥1) | Disappear (0.1% & ≥2) |
|---|---|---|---|---|
| **Introduced** | | | | |
| *Adlercreutzia* | ✓ | – | – | – |
| *Allisonella* | – | ✓ | – | – |
| *Anaerobiospirillum* | ✓ | – | – | – |
| *Anaerococcus* | ✓ | – | – | – |
| *Anaerofilum* | ✓ | – | – | – |
| *Anaerofustis* | ✓ | – | – | – |
| *Angelakisella* | ✓ | – | – | – |
| *Bilophila* | ✓ | – | – | – |
| *Carnobacterium* | ✓ | – | – | – |
| *Cellulosilyticum* | ✓ | – | – | – |
| *Christensenella* | ✓ | – | – | – |
| *Citrobacter* | ✓ | – | – | – |
| *Clostridium sensu stricto 5* | ✓ | – | – | – |
| *Coprobacillus* | ✓ | ✓ | – | – |
| *Coprobacter* | – | ✓ | – | – |
| *Corynebacterium* | ✓ | – | – | – |
| *Dialister* | ✓ | – | – | – |
| *Eisenbergiella* | ✓ | ✓ | – | – |
| *Enterobacter* | ✓ | – | – | – |
| *Epulopiscium* | ✓ | – | – | – |
| *Eremococcus* | ✓ | – | – | – |
| *Escherichia-Shigella* | ✓ | ✓ | – | – |
| *Faecalitalea* | ✓ | – | – | – |
| *Family XIII UCG-001* | – | ✓ | – | – |
| *Finegoldia* | – | ✓ | – | – |
| *Flavonifractor* | – | ✓ | – | – |
| *Fusobacterium* | – | ✓ | – | – |
| *GCA-900066755* | ✓ | – | – | – |
| *Howardella* | – | ✓ | – | – |
| *Hungatella* | ✓ | ✓ | – | – |
| *Klebsiella* | ✓ | – | – | – |

**Supplementary Table S3. Genera appearing or disappearing during the Arctic diet phase under permissive and strict calling rules.**

| **Genus** | Appear (no thr, ≥1) | Appear (0.1% & ≥2) | Disappear (no thr, ≥1) | Disappear (0.1% & ≥2) |
|---|---|---|---|---|
| *Lachnospiraceae UCG-008* | ✓ | – | – | – |
| *Lachnospiraceae UCG-009* | ✓ | – | – | – |
| *Lachnospiraceae UCG-010* | – | ✓ | – | – |
| *Lactonifactor* | ✓ | – | – | – |
| *Latilactobacillus* | ✓ | – | – | – |
| *Lawsonella* | ✓ | – | – | – |
| *Leuconostoc* | ✓ | – | – | – |
| *Mailhella* | – | ✓ | – | – |
| *Megamonas* | ✓ | – | – | – |
| *Methanobrevibacter* | ✓ | – | – | – |
| *Negativibacillus* | – | ✓ | – | – |
| *Neisseria* | ✓ | – | – | – |
| *Oscillibacter* | – | ✓ | – | – |
| *Oxalobacter* | ✓ | – | – | – |
| *Paeniclostridium* | ✓ | ✓ | – | – |
| *Parvibacter* | ✓ | – | – | – |
| *Peptoniphilus* | ✓ | – | – | – |
| *Peptostreptococcus* | ✓ | – | – | – |
| *Photobacterium* | ✓ | ✓ | – | – |
| *Prevotella* | ✓ | ✓ | – | – |
| *Raoultibacter* | ✓ | – | – | – |
| *Robinsoniella* | ✓ | ✓ | – | – |
| *Sanguibacteroides* | – | ✓ | – | – |
| *Sellimonas* | ✓ | – | – | – |
| *Senegalimassilia* | – | ✓ | – | – |
| *Slackia* | – | ✓ | – | – |
| *Staphylococcus* | ✓ | – | – | – |
| *Turicibacter* | – | ✓ | – | – |
| *Tuzzerella* | – | ✓ | – | – |
| *UBA1819* | – | ✓ | – | – |
| *[Eubacterium] fissicatena group* | ✓ | – | – | – |
| *[Eubacterium] nodatum group* | ✓ | ✓ | – | – |

## Supplementary Table S3. Genera appearing or disappearing during the Arctic diet phase under permissive and strict calling rules.

| Genus | Appear (no thr, ≥1) | Appear (0.1% & ≥2) | Disappear (no thr, ≥1) | Disappear (0.1% & ≥2) |
|---|---|---|---|---|
| *[Ruminococcus] gnavus group* | ✓ | ✓ | – | – |
| **Disappeared** | | | | |
| *Agathobacter* | – | – | – | ✓ |
| *Bifidobacterium* | – | – | – | ✓ |
| *DTU089* | – | – | ✓ | – |
| *Hafnia-Obesumbacterium* | – | – | ✓ | ✓ |
| *Lachnospiraceae UCG-001* | – | – | ✓ | ✓ |
| *Oribacterium* | – | – | ✓ | – |
| *Papillibacter* | – | – | ✓ | – |
| *Paraprevotella* | – | – | ✓ | ✓ |
| *Prevotella 9* | – | – | – | ✓ |
| *Prevotellaceae UCG-001* | – | – | ✓ | ✓ |
| *[Bacteroides] pectinophilus group* | – | – | ✓ | – |
| *[Eubacterium] siraeum group* | – | – | ✓ | – |

*Genera were evaluated under two calling rules: permissive (no threshold, detected in ≥ 1 sample) and strict (≥0.1% relative abundance in ≥ 2 samples). Check marks indicate that a genus met the corresponding appearance or disappearance criterion under the specified rule. Some genera were classified differently depending on the rule applied.*
