# Supplementary material for: Dietary transition to an Indigenous Greenlandic diet induces instant shifts in gut microbiota composition – a pilot intervention study
Source: Front Microbiomes. 2026 May 21;5:1832705. doi: 10.3389/frmbi.2026.1832705 (PMC13234626; doi:10.3389/frmbi.2026.1832705)
Supplement: Supplementary file 8 [file Table4.pdf]

Supplementary Table 4: Pairwise Wilcoxon rank sum tests for selected genera across dietary phases.

**Genus**	UK before vs Arctic diet	Arctic diet vs UK after	UK before vs UK after
Animal-associated			
Alistipes	raw p = 0.0066 | adj p = 0.0198 (*)	raw p = 0.3387 | adj p = 0.3387 (ns)	raw p = 0.1116 | adj p = 0.1674 (ns)
Bacteroides	raw p = 0.0134 | adj p = 0.0401 (*)	raw p = 0.0353 | adj p = 0.0530 (ns)	raw p = 0.1116 | adj p = 0.1116 (ns)
Intestinimonas	raw p = 0.0024 | adj p = 0.0071(**)	raw p = 0.0691 | adj p = 0.1036 (ns)	raw p = 0.1116 | adj p = 0.1116 (ns)
Lachnoclostridium	raw p = 0.0024 | adj p = 0.0071(**)	raw p = 0.3892 | adj p = 0.3892 (ns)	raw p = 0.3768 | adj p = 0.3892 (ns)
Plant-associated			
Faecalibacterium	raw p = 0.0066 | adj p = 0.0198 (*)	raw p = 0.0691 | adj p = 0.1036 (ns)	raw p = 0.8597 | adj p = 0.8597 (ns)
Lachnospira	raw p = 0.0027 | adj p = 0.0080 (**)	raw p = 0.0563 | adj p = 0.0844 (ns)	raw p = 0.2159 | adj p = 0.2159 (ns)
Prevotella total	raw p = 0.0024 | adj p = 0.0071 (**)	raw p = 0.0217 | adj p = 0.0325 (*)	raw p = 0.1116 | adj p = 0.1116 (ns)
Ruminococcus	raw p = 0.0059 | adj p = 0.0176 (*)	raw p = 0.0151 | adj p = 0.0226 (*)	raw p = 1.0000 | adj p = 1.0000 (ns)
Prevotella clades vs Prevotella combined			
Prevotella	raw p = 0.1790 | adj p = 0.5371 (ns)	raw p = 0.7392 | adj p = 0.7392 (ns)	raw p = 0.3865 | adj p = 0.5797 (ns)
Prevotella 7	raw p = 0.3106 | adj p = 0.3106 (ns)	raw p = 0.0217 | adj p = 0.0650 (ns)	raw p = 0.2159 | adj p = 0.3106 (ns)
Prevotella 9	raw p = 0.0006 | adj p = 0.0017 (**)	raw p = 0.2031 | adj p = 0.2031 (ns)	raw p = 0.0518 | adj p = 0.0777 (ns)
Prevotella total	raw p = 0.0024 | adj p = 0.0071 (**)	raw p = 0.0217 | adj p = 0.0325 (*)	raw p = 0.1116 | adj p = 0.1116 (ns)

Raw and Benjamini-Hochberg-adjusted p-values for comparison between UK diet before, Arctic diet, and UK diet after. Genera are grouped into those associated with protein- and fat-rich dietary patterns, those associated with fiber-rich dietary patterns, and Prevotella clades. Wilcoxon rank-sum tests were performed separately for each genus, and Benjamini-Hochberg correction was applied across the three pairwise comparisons within each genus.
